# Timoshenko–Ehrenfest Beam‐Based Reconfigurable Elastic Metasurfaces for Multifunctional Wave Manipulation

**DOI:** 10.1002/advs.202400090

**Published:** 2024-03-14

**Authors:** Geon Lee, Wonjae Choi, Bonggyu Ji, Miso Kim, Junsuk Rho

**Affiliations:** ^1^ Department of Mechanical Engineering Pohang University of Science and Technology (POSTECH) Pohang 37673 Republic of Korea; ^2^ Intelligent Wave Engineering Team Korea Research Institute of Standards and Science (KRISS) Daejeon 34113 Republic of Korea; ^3^ Department of Precision Measurement University of Science and Technology (UST) Daejeon 34113 Republic of Korea; ^4^ Korea Automotive Tuning Institute of Safety Technology Testing Certification Office, Korea Transportation Safety Authority (KOTSA) Gimcheon 39506 Republic of Korea; ^5^ School of Advanced Materials Science and Engineering Sungkyunkwan University (SKKU) Suwon 16519 Republic of Korea; ^6^ SKKU Institute of Energy Science and Engineering (SIEST) Sungkyunkwan University (SKKU) Suwon 16519 Republic of Korea; ^7^ Department of Chemical Engineering Pohang University of Science and Technology (POSTECH) Pohang 37673 Republic of Korea; ^8^ Department of Electrical Engineering Pohang University of Science and Technology (POSTECH) Pohang 37673 Republic of Korea; ^9^ POSCH‐POSTECH‐RIST Convergence Research Center for Flat Optics and Metaphotonics Pohang 37673 Republic of Korea

**Keywords:** elastic metasurface, generalized Snell's law, reconfigurability and multifunctionality, Timoshenko–Ehrenfest beam theory, transfer matrix method

## Abstract

Herein, a Timoshenko–Ehrenfest beam‐based reconfigurable elastic metasurface is introduced that can perform multifunctional wave phenomena within a single substrate, featuring high transmission in the ultrabroadband frequency range. Conventional elastic metasurfaces are typically limited to specific purposes and frequencies, thereby imposing significant constraints on their practical application. The approach involves assembly‐components with various geometries on a substrate for reconfigurability, enabling to easily control and implement multifunctional wave phenomena, including anomalous‐refraction, focusing, self‐acceleration, and total‐reflection. This is the first study on elastic metasurfaces to theoretically analyze the dispersion relation based on the Timoshenko–Ehrenfest beam theory, which considers shear deformations and rotational inertia. The analytical model is validated by demonstrating an excellent agreement with numerical and experimental results. The findings include full‐wave harmonic simulations and experimentally visualized fields for measuring various wave modulations. Furthermore, the practicality of the system is verified by significantly enhancing the piezoelectric energy harvesting performance within the focusing configuration. It is believed that the reconfigurable elastic metasurface and analytical model based on the Timoshenko–Ehrenfest beam theory have vast applications such as structural health monitoring, wireless sensing, and Internet of Things.

## Graphical abstract

A Timoshenko–Ehrenfest beam‐based reconfigurable elastic metasurface (TREM) is explored for multifunctional wave modulation. The metasurface is reconfigured by replacing assembly‐components with various heights to impart distinct phase‐shift abilities. The unit cell of the metasurface is analytically investigated based on the Timoshenko–Ehrenfest beam theory. Additionally, to verify the practicability of the metasurface, highly amplified piezoelectric energy harvesting performance is achieved in the focusing configuration.

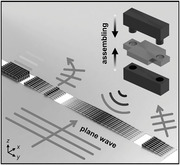

## 1 Introduction

Metamaterials^[^ ^1^, ^2^, ^3^ ^]^ configured by the periodic arrangement of unit cells have drawn a significant interest from the scientific community owing to their extraordinary ability to realize wave characteristics that are not observable in nature. Nevertheless, 3D metamaterials encountered practical challenges owing to their wavelength‐dependent size and the intricate coupling of various wave modes, making practical applications difficult. To address these limitations, metasurfaces, which are reduced‐dimensional forms of metamaterials, have been used in diverse wave domains such as photonics^[^ ^4^, ^5^, ^6^ ^]^ and acoustics.^[^ ^7^, ^8^, ^9^ ^]^ A metasurface is a tailored arrangement of subwavelength unit cells to control waves, allowing for modulation of their amplitude, phase, and polarization properties. More recently, studies on elastic metasurfaces have been actively underway to achieve unconventional elastic wave modulation, such as anomalous refraction, focus, self‐acceleration, and full reflection.^[^ ^10^, ^11^, ^12^, ^13^ ^]^ Despite the potential for various wave manipulations using elastic metasurfaces, most previous studies have significant practical constraints because the designed passive elastic metasurfaces typically serve a single function or operate within a specific frequency range.

Metasurfaces are designed based on the correlation between lattice constants and working wavelengths; the wavelengths of elastic waves range from millimeters to meters. Hence, once cumbersome structures are fabricated, tailoring the functionality or working frequency is challenging. Therefore, reconfigurable metasurfaces that can implement various wave phenomena on a single platform should be explored across all wave‐related disciplines. Two representative wave‐modulation techniques exist: using responses induced by other stimuli and reconfiguring mechanical structures. Various methods exist for controlling the wavefront in elastic metasurfaces using stimuli from different sources, primarily involving the use of electric or magnetic fields. First, a programmable elastic metasurface incorporating sensing and actuating unit cells made of piezoelectric materials was introduced to enable the real‐time multifunctional control of elastic waves from an external electrical circuit.^[^ ^14^ ^]^ Subsequently, an active‐coding elastic metasurface comprising stacked piezoelectric patches was developed by adjusting negative capacitance.^[^ ^15^ ^]^ Subsequently, an adaptive elastic metasurface was proposed, tuning negative capacitance effectively adjusting Young's modulus.^[^ ^16^ ^]^ Conversely, by harnessing the power of a magnetic field, a magneto‐elastic metasurface was proposed, considering the magneto‐mechanical coupling of magnetostrictive materials through a magnetic field and pre‐stress adjustments.^[^ ^17^, ^18^ ^]^ However, unlike light^[^ ^19^, ^20^ ^]^ or electromagnetic^[^ ^21^, ^22^ ^]^ waves, inducing electromagnetic or chemical responses from elastic waves using a bias field is challenging. Moreover, tuning the phase‐shift for each transducer separately within the phased array introduces complexities and incurs high costs for the operational system. Furthermore, considering that the externally biased force does not linearly vary with the size of the structure, it poses challenges in applying it to various sizes of applications. On the other hand, elastic metasurfaces designed based on linear elastic dynamics possess the advantage of easy mechanical reconfiguration owing to their manageable size. To leverage this advantage, Lin et al.^[^ ^23^ ^]^ study proposed the phase control of elastic waves by varying the geometric parameters of a mass oscillator. Subsequently, the phase‐shift of the elastic wave can span the full‐phase range by adjusting the distance between the mass oscillators.^[^ ^24^ ^]^ Furthermore, a reconfigurable fishbone elastic metasurface that enables continuous tuning by screwing in or out to achieve switchable multifunctional elastic wave manipulation was proposed.^[^ ^25^, ^26^, ^27^ ^]^

However, the most intriguing reconfigurable elastic metasurfaces introduced earlier have significant limitations. First, an analytical approach is necessary for a comprehensive physical understanding and predicting the propagating behaviors of elastic waves. Still, most prior studies have predominantly relied on numerical analysis.^[^ ^17^, ^18^, ^23^, ^24^, ^25^, ^26^, ^27^, ^28^ ^]^ This is because the proposed geometries for the phase modulation of elastic waves are highly complex and challenging to model mathematically. Second, because of the complexity of such systems, experimental validations have been challenging, leading to a substantial number of studies reporting without experimental validations.^[^ ^15^, ^16^, ^17^, ^23^, ^24^, ^26^ ^]^

In this study, for the first time, we proposed a comprehensive analysis combining theoretical, numerical, and experimental approaches to explore a new type of Timoshenko–Ehrenfest beam‐based reconfigurable elastic metasurface (TREM) for the multifunctional manipulation of elastic waves, particularly flexural waves. We analyzed the assembled unit cells using an analytical framework based on the Timoshenko–Ehrenfest beam theory, which overcomes the limitations of the conventional Euler–Bernoulli beam theory. A transfer matrix method was employed to integrate the individual beam sections to configure the unit cells of the TREM. Under the assumption of infinitely extended boundary conditions, we analytically derived dispersion relations to facilitate a thorough analysis of various wave phenomena. Furthermore, our mathematical modeling process was employed to support other analyses. We numerically and experimentally validated multifunctional wave phenomena, including anomalous refraction, wave focusing, self‐acceleration, and total reflection, based on the generalized Snell's law^[^ ^29^ ^]^ by reconfiguring assembly‐components on a single‐substrate platform while maintaining a high transmission ratio over an ultrabroadband frequency range. Additionally, we explored the conversion of focused elastic wave energy into piezoelectric energy, confirming the feasibility of our proposed reconfigurable TREM for various application scenarios, such as the Internet of Things, structural health monitoring, and signal processing.

## 2 Design Strategy of TREM

### 2.1 Unit Cell Design

**Figure 1:**
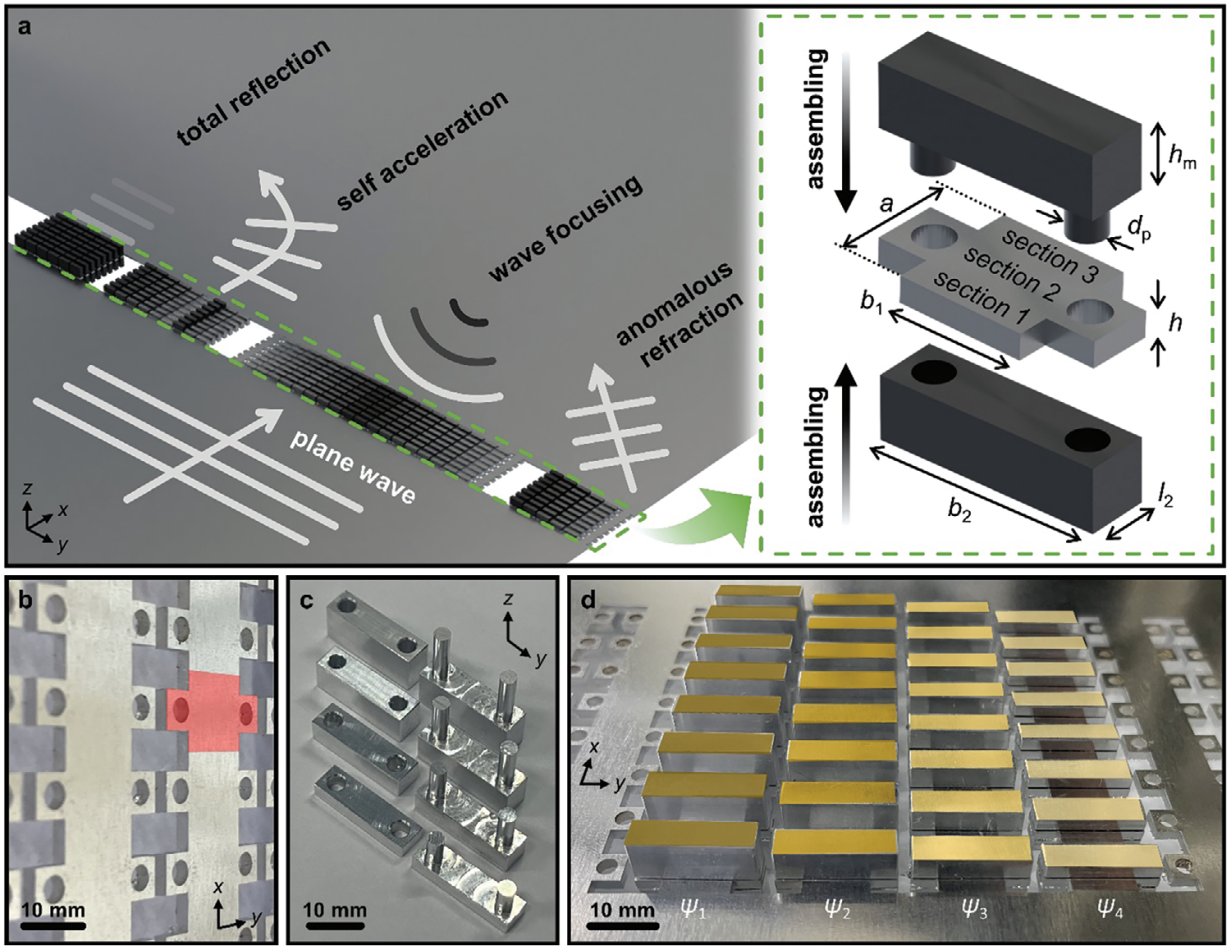
a) Schematic of the Timoshenko–Ehrenfest beam‐based reconfigurable elastic metasurface (TREM) for the multifunctional wave manipulation and the corresponding unit cell assembling process. b) Picture of the aluminum thin plate, which is the substrate for the c) assembly‐components with various values of *h*_m_. The red‐shaded region represents the unit cell of TREM. d) Picture showing the assembled TREM on the substrative plate. Each meta‐slab is configured to control the individual phase‐shift *ψ*.

To investigate multifunctional wave physics using an elastic metasurface, the full phase‐shift covering condition must be satisfied by adjusting the geometric parameters. However, such complex designs have limitations in the analytical modeling of metasurface unit cells. Therefore, in this study, we propose a simple but accurate method to manipulate the phase‐shift and achieve reconfigurability. The proposed unit cell of TREM was designed with three irreducible beam sections with various cross‐sectional areas (**Figure** **1**a). The cross‐sectional area of the second beam section can be adjusted by coupling assembly‐components. The assembly‐components form pairs, where the pillar of the male‐component *d*_p_ penetrates the thin plate and forms a unit cell by engaging with the female‐component with a hole of the identical diameter. A thin plate, serving as a medium for propagating elastic waves, was arranged with unit cells comprising the meta‐slabs (Figure 1b). The proposed TREM controls phase‐shift by combining assembly‐components with different values of *h*_m_, which control the dispersive properties of the propagating elastic waves by introducing additional locally resonating oscillators (Figure 1c).^[^ ^30^, ^31^ ^]^ Then, the interconnected meta‐slab with assembly‐components possessed individual phase‐shifting capabilities for each meta‐slab and was used for multifunctional wavefront manipulation through their configuration such as anomalous refraction, focusing, self‐acceleration, and total reflection (Figure 1d). The multifunctional control of these features can be easily reconfigurable by replacing only assembly‐components on a fixed plate substrate. All components used in this study were fabricated using conventional aluminum, and the exploration of different dispersive properties can be achieved by employing alternative materials.

**Figure 2:**
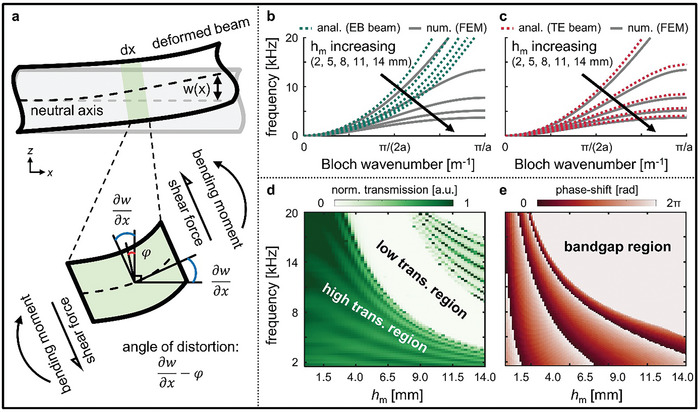
a) Schematic representing the Timoshenko–Ehrenfest (TE) beam theory, used for the theoretical analysis of reconfigurable elastic metasurface. Dispersion curves based on the b) Euler–Bernoulli (EB) beam theory and c) TE beam theory compared with numerically calculated results using the finite element method (FEM) by varying *h*_m_. d) Frequency spectrum of transmission and e) phase‐shift for various *h*_m_.

To analyze the dynamic behavior of each beam section theoretically, there are two representative governing equations: the Euler–Bernoulli beam theory^[^ ^32^, ^33^ ^]^ and the Timoshenko–Ehrenfest beam theory.^[^ ^34^, ^35^ ^]^ However, when designing elastic metasurfaces, the Euler–Bernoulli beam theory causes significant errors because of the low slenderness ratio of the unit cell. Metasurfaces are inevitably composed of a low slenderness ratio for satisfying subwavelength‐scale conditions where the working wavelength is smaller than the periodicity of the unit cell and varying the geometrical parameters to achieve a full phase‐shift. For these reasons, we adopted the Timoshenko–Ehrenfest beam theory as the governing equation that additionally considers shear deformation and rotational inertia to analyze our proposed beam‐based unit cell with a low slenderness ratio (**Figure** **2**a). To model the dynamic Timoshenko–Ehrenfest beam, we started with the Euler–Lagrange equations, which can be derived by applying Hamilton's principle as follows:^[^ ^36^, ^37^ ^]^

(1a) $$\rho_{i}S_{i}\frac{\partial^{2}w_{i}\left(x,t\right)}{\partial t^{2}}-\frac{\partial }{\partial x}\left[\kappa_{i}G_{i}S_{i}\left\{\frac{\partial w_{i}\left(x,t\right)}{\partial x}-\varphi_{i}\left(x,t\right)\right\}\right]=\;0$$

(1b) $$\begin{array}{ccc} \rho_{i}I_{i}\frac{\partial^{2}\varphi_{i}\left(x,t\right)}{\partial t^{2}} & & -\frac{\partial }{\partial x}\left\{E_{i}I_{i}\frac{\partial \varphi_{i}\left(x,t\right)}{\partial x}\right\}\\ & & -\;\;\kappa_{i}G_{i}S_{i}\left\{\frac{\partial w_{i}\left(x,t\right)}{\partial x}-\varphi_{i}\left(x,t\right)\right\}=\;0 \end{array}$$

where *ρ*_*i*_, *E*_*i*_, and *G*_*i*_ represent the mass density, Young's modulus, and shear modulus of each section, which was determined by the material properties, respectively. *S*_*i*_, *I*_*i*_, and *κ*_*i*_ represent the cross‐sectional area, second moment of the area, and Timoshenko shear coefficient of each section, which was determined by the geometric parameters, respectively. For linear elastic, isotropic, and homogeneous beams, the equations of motion are derived as follows:

(2a) $$\begin{array}{ccc} E_{i}I_{i}\frac{\partial^{4}w_{i}\left(x,t\right)}{\partial x^{4}} & & +\;\;\rho_{i}S_{i}\frac{\partial^{2}w_{i}\left(x,t\right)}{\partial t^{2}}-\rho_{i}I_{i}\left(1+\frac{E_{i}}{\kappa_{i}G_{i}}\right)\frac{\partial^{4}w_{i}\left(x,t\right)}{\partial t^{2}\partial x^{2}}\\ & & +\;\left(\frac{\rho_{i}^{2}I_{i}}{\kappa_{i}G_{i}}\right)\frac{\partial^{4}w_{i}\left(x,t\right)}{\partial t^{4}}=\;0 \end{array}$$

(2b) $$\begin{array}{ccc} E_{i}I_{i}\frac{\partial^{4}\varphi_{i}\left(x,t\right)}{\partial x^{4}} & & +\;\;\rho_{i}S_{i}\frac{\partial^{2}\varphi_{i}\left(x,t\right)}{\partial t^{2}}-\rho_{i}I_{i}\left(1+\frac{E_{i}}{\kappa_{i}G_{i}}\right)\frac{\partial^{4}\varphi_{i}\left(x,t\right)}{\partial t^{2}\partial x^{2}}\\ & & +\;\left(\frac{\rho_{i}^{2}I_{i}}{\kappa_{i}G_{i}}\right)\frac{\partial^{4}\varphi_{i}\left(x,t\right)}{\partial t^{4}}=\;0 \end{array}$$

Using the method of undetermined coefficients, separable solutions are defined as:

(3a) $$w_{i}\left(x,t\right)=W_{i}\left(x\right)e^{j\omega t}$$

(3b) $$\varphi_{i}\left(x,t\right)=\Phi_{i}\left(x\right)e^{j\omega t}$$

where *W*_*i*_, *Φ*_*i*_, and *ω* represent the transverse vibration mode, rotational mode, and angular frequency, respectively, and $j\;=\sqrt{-1}\;$. By substituting Equations 2 and 3, we obtain the following fourth‐order ordinary differential equation:

(4a) $$W_{i}^{\prime \prime \prime \prime }\left(x\right)+\left(\alpha_{i}+\beta_{i}\right)W_{i}^{\prime \prime }\left(x\right)+\left(\alpha_{i}\beta_{i}-\gamma_{i}\right)W_{i}\left(x\right)=\;0$$

(4b) $$\Phi_{i}^{\prime \prime \prime \prime }\left(x\right)+\left(\alpha_{i}+\beta_{i}\right)\Phi_{i}^{\prime \prime }\left(x\right)+\left(\alpha_{i}\beta_{i}-\gamma_{i}\right)\Phi_{i}\left(x\right)=\;0$$

where,

(5a) $$\alpha_{i}=\left(\frac{\rho_{i}}{E_{i}}\right)\omega^{2}$$

(5b) $$\beta_{i}=\left(\frac{\rho_{i}}{\kappa_{i}G_{i}}\right)\omega^{2}$$

(5c) $$\gamma_{i}=\left(\frac{\rho_{i}S_{i}}{E_{i}I_{i}}\right)\omega^{2}$$

The general solutions can be obtained by assuming that $W_{i}\left(x\right)=e^{k_{\left(i\right)}x}\;$ as follows:

(6a) $$W_{i}\left(x\right)=A_{i}e^{k_{1\left(i\right)}l_{i}}+B_{i}e^{-k_{1\left(i\right)}l_{i}}+C_{i}e^{k_{2\left(i\right)}l_{i}}+D_{i}e^{-k_{2\left(i\right)}l_{i}}$$

(6b) $$\begin{array}{ccc} \Phi_{i}\left(x\right) & = & \left(\frac{\beta_{i}}{k_{1\left(i\right)}}+k_{1\left(i\right)}\right)A_{i}e^{k_{1\left(i\right)}l_{i}}-\left(\frac{\beta_{i}}{k_{1\left(i\right)}}+k_{1\left(i\right)}\right)B_{i}e^{-k_{1\left(i\right)}l_{i}}\\ & & +\left(\frac{\beta_{i}}{k_{2\left(i\right)}}+k_{2\left(i\right)}\right)C_{i}e^{k_{2\left(i\right)}l_{i}}-\left(\frac{\beta_{i}}{k_{2\left(i\right)}}+k_{2\left(i\right)}\right)D_{i}e^{-k_{2\left(i\right)}l_{i}} \end{array}$$

where *A*_*i*_, *B*_*i*_, *C*_*i*_, and *D*_*i*_ are coefficients, and

(7a) $$l_{i}=\;x-x_{i-1}$$

(7b) $$k_{1\left(i\right)}={\left[-\left(\frac{\alpha_{i}+\beta_{i}}{2}\right)+\frac{{\left\{{\left(\alpha_{i}-\beta_{i}\right)}^{2}+4\gamma_{i}\right\}}^{\frac{1}{2}}}{2}\right]}^{\frac{1}{2}}\;$$

(7c) $$k_{2\left(i\right)}={\left[-\left(\frac{\alpha_{i}+\beta_{i}}{2}\right)-\frac{{\left\{{\left(\alpha_{i}-\beta_{i}\right)}^{2}+4\gamma_{i}\right\}}^{\frac{1}{2}}}{2}\right]}^{\frac{1}{2}}\;$$

To connect each adjacent beam section of the unit cell, we applied the compatibility conditions:

(8a) $$W_{i}\left(x_{i}^{-}\right)=W_{i+1}\left(x_{i}^{+}\right)$$

(8b) $$\Phi_{i}\left(x_{i}^{-}\right)=\Phi_{i+1}\left(x_{i}^{+}\right)$$

(8c) $$E_{i}I_{i}\Phi_{i}^{\prime }\left(x_{i}^{-}\right)=E_{i+1}I_{i+1}\Phi_{i+1}^{\prime }\left(x_{i}^{+}\right)$$

(8d) $$\begin{array}{c} \kappa_{i}G_{i}S_{i}\left\{W_{i}^{\prime }\left(x_{i}^{-}\right)-\Phi_{i}\left(x_{i}^{-}\right)\right\}=\kappa_{i+1}G_{i+1}S_{i+1}\\ \left\{W_{i+1}^{\prime }\left(x_{i}^{+}\right)-\Phi_{i+1}\left(x_{i}^{+}\right)\right\} \end{array}$$

where each equation represents the compatibility conditions for the displacement, slope, bending moment, and shear force between the adjacent beams. Therefore, we derived a transfer matrix ***T***_*i*,*i* + 1(*i*)_ that connects the adjacent *i^th^* and (*i* + 1)^*th*^ beam sections as follows:

(9a) $$T_{i,i+1\left(i\right)}{\left[\begin{array}{c} A\\ B\\ C\\ D \end{array}\right]}_{i}=T_{i,i+1\left(i+1\right)}{\left[\begin{array}{c} A\\ B\\ C\\ D \end{array}\right]}_{i+1}$$

(9b) $${\left[\begin{array}{c} A\\ B\\ C\\ D \end{array}\right]}_{i}=T_{i,i+1\left(i\right)}^{-1}T_{i,i+1\left(i+1\right)}T_{i+1,i+2\left(i+1\right)}^{-1}T_{i+1,i+2\left(i+2\right)}{\left[\begin{array}{c} A\\ B\\ C\\ D \end{array}\right]}_{i+2}$$

where,

(10a) $$T_{i,i+1\left(i\right)}=\left[\begin{array}{cccc} e^{k_{1\left(i\right)}l_{i}} & e^{-k_{1\left(i\right)}l_{i}} & e^{k_{2\left(i\right)}l_{i}} & e^{-k_{2\left(i\right)}l_{i}}\\ \left(\frac{\beta_{i}+k_{1\left(i\right)}^{2}}{k_{1\left(i\right)}}\right)e^{k_{1\left(i\right)}l_{i}} & -\left(\frac{\beta_{i}+k_{1\left(i\right)}^{2}}{k_{1\left(i\right)}}\right)e^{-k_{1\left(i\right)}l_{i}} & \left(\frac{\beta_{i}+k_{2\left(i\right)}^{2}}{k_{2\left(i\right)}}\right)e^{k_{2\left(i\right)}l_{i}} & -\left(\frac{\beta_{i}+k_{2\left(i\right)}^{2}}{k_{2\left(i\right)}}\right)e^{-k_{2\left(i\right)}l_{i}}\\ E_{i}I_{i}\left(\beta_{i}+k_{1\left(i\right)}^{2}\right)e^{k_{1\left(i\right)}l_{i}} & E_{i}I_{i}\left(\beta_{i}+k_{1\left(i\right)}^{2}\right)e^{-k_{1\left(i\right)}l_{i}} & E_{i}I_{i}\left(\beta_{i}+k_{2\left(i\right)}^{2}\right)e^{k_{2\left(i\right)}l_{i}} & E_{i}I_{i}\left(\beta_{i}+k_{2\left(i\right)}^{2}\right)e^{-k_{2\left(i\right)}l_{i}}\\ -\frac{\kappa_{i}G_{i}S_{i}\beta_{i}}{k_{1\left(i\right)}}e^{k_{1\left(i\right)}l_{i}} & \frac{\kappa_{i}G_{i}S_{i}\beta_{i}}{k_{1\left(i\right)}}e^{-k_{1\left(i\right)}l_{i}} & -\frac{\kappa_{i}G_{i}S_{i}\beta_{i}}{k_{2\left(i\right)}}e^{k_{2\left(i\right)}l_{i}} & \frac{\kappa_{i}G_{i}S_{i}\beta_{i}}{k_{2\left(i\right)}}e^{-k_{2\left(i\right)}l_{i}} \end{array}\right]$$

(10b) $$T_{i,i+1\left(i+1\right)}=\left[\begin{array}{cccc} 1 & 1 & 1 & 1\\ \frac{\beta_{i+1}+k_{1\left(i+1\right)}^{2}}{k_{1\left(i+1\right)}} & -\frac{\beta_{i+1}+k_{1\left(i+1\right)}^{2}}{k_{1\left(i+1\right)}} & \frac{\beta_{i+1}+k_{2\left(i+1\right)}^{2}}{k_{2\left(i+1\right)}} & -\frac{\beta_{i+1}+k_{2\left(i+1\right)}^{2}}{k_{2\left(i+1\right)}}\\ E_{i+1}I_{i+1}\left(\beta_{i+1}+k_{1\left(i+1\right)}^{2}\right) & E_{i+1}I_{i+1}\left(\beta_{i+1}+k_{1\left(i+1\right)}^{2}\right) & E_{i+1}I_{i+1}\left(\beta_{i+1}+k_{2\left(i+1\right)}^{2}\right) & E_{i+1}I_{i+1}\left(\beta_{i+1}+k_{2\left(i+1\right)}^{2}\right)\\ -\frac{\kappa_{i+1}G_{i+1}S_{i+1}\beta_{i+1}}{k_{1\left(i+1\right)}} & \frac{\kappa_{i+1}G_{i+1}S_{i+1}\beta_{i+1}}{k_{1\left(i+1\right)}} & -\frac{\kappa_{i+1}G_{i+1}S_{i+1}\beta_{i+1}}{k_{2\left(i+1\right)}} & \frac{\kappa_{i+1}G_{i+1}S_{i+1}\beta_{i+1}}{k_{2\left(i+1\right)}} \end{array}\right]\;$$

### 2.2 Frequency Dispersion Relation

Next, to investigate the wave physics of the infinitely arrayed unit cells, we applied the Floquet–Bloch boundary conditions at the ends of the unit cells as follows:^[^ ^38^ ^]^

(11a) $$W_{i}\left(0^{+}\right)e^{\mathrm{jka}}=W_{i+2}\left(a^{-}\right)$$

(11b) $$\Phi_{i}\left(0^{+}\right)e^{\mathrm{jka}}=\Phi_{i+2}\left(a^{-}\right)$$

(11c) $$E_{i}I_{i}\Phi_{i}^{\prime }\left(0^{+}\right)e^{\mathrm{jka}}=E_{i+2}I_{i+2}\Phi_{i+2}^{\prime }\left(a^{-}\right)$$

(11d) $$\begin{array}{ccc} & & \kappa_{i}G_{i}S_{i}\left\{W_{i}^{\prime }\left(0^{+}\right)-\Phi_{i}\left(0^{+}\right)\right\}e^{\mathrm{jka}}=\kappa_{i+2}G_{i+2}S_{i+2}\left\{W_{i+2}^{\prime }\left(a^{-}\right)\right.\\ & & \left.-\Phi_{i+2}\left(a^{-}\right)\right\} \end{array}$$

where *a*  = *l*_1_  + *l*_2_ + *l*_3_ is the lattice constant of the unit cell and *k* is the 1D Bloch wavenumber. Therefore, we derived boundary matrix ***B***_*i*,*i* + 2(*i*)_ that connects the adjacent *i^th^* and (*i* + 2)^*th*^ periodic unit beam sections.

(12a) $$B_{i,i+2\left(i\right)}{\left[\begin{array}{c} A\\ B\\ C\\ D \end{array}\right]}_{i}=e^{-\mathrm{jka}}B_{i,i+2\left(i+2\right)}{\left[\begin{array}{c} A\\ B\\ C\\ D \end{array}\right]}_{i+2}$$

(12b) $${\left[\begin{array}{c} A\\ B\\ C\\ D \end{array}\right]}_{i}=B_{i,i+2\left(i\right)}^{-1}e^{-\mathrm{jka}}B_{i,i+2\left(i+2\right)}{\left[\begin{array}{c} A\\ B\\ C\\ D \end{array}\right]}_{i+2}$$

where,

(13a) $$B_{i,i+2\left(i\right)}=\left[\begin{array}{cccc} 1 & 1 & 1 & 1\\ \frac{\beta_{i}+k_{1\left(i\right)}^{2}}{k_{1\left(i\right)}} & -\frac{\beta_{i}+k_{1\left(i\right)}^{2}}{k_{1\left(i\right)}} & \frac{\beta_{i}+k_{2\left(i\right)}^{2}}{k_{2\left(i\right)}} & -\frac{\beta_{i}+k_{2\left(i\right)}^{2}}{k_{2\left(i\right)}}\\ E_{i}I_{i}\left(\beta_{i}+k_{1\left(i\right)}^{2}\right) & E_{i}I_{i}\left(\beta_{i}+k_{1\left(i\right)}^{2}\right) & E_{i}I_{i}\left(\beta_{i}+k_{2\left(i\right)}^{2}\right) & E_{i}I_{i}\left(\beta_{i}+k_{2\left(i\right)}^{2}\right)\\ -\frac{\kappa_{i}G_{i}S_{i}\beta_{i}}{k_{1\left(i\right)}} & \frac{\kappa_{i}G_{i}S_{i}\beta_{i}}{k_{1\left(i\right)}} & -\frac{\kappa_{i}G_{i}S_{i}\beta_{i}}{k_{2\left(i\right)}} & \frac{\kappa_{i}G_{i}S_{i}\beta_{i}}{k_{2\left(i\right)}} \end{array}\right]\;$$

(13b) $$\begin{array}{ccc} & & \;\;B_{i,i+2\left(i+2\right)}\\ & & \;\;=\left[\begin{array}{cccc} e^{k_{1\left(i+2\right)}l_{i+2}} & e^{-k_{1\left(i+2\right)}l_{i+2}} & e^{k_{2\left(i+2\right)}l_{i+2}} & e^{-k_{2\left(i+2\right)}l_{i+2}}\\ \left(\frac{\beta_{i+1}+k_{1\left(i+1\right)}^{2}}{k_{1\left(i+1\right)}}\right)e^{k_{1\left(i+2\right)}l_{i+2}} & -\left(\frac{\beta_{i+1}+k_{1\left(i+1\right)}^{2}}{k_{1\left(i+1\right)}}\right)e^{-k_{1\left(i+2\right)}l_{i+2}} & \left(\frac{\beta_{i+1}+k_{2\left(i+1\right)}^{2}}{k_{2\left(i+1\right)}}\right)e^{k_{2\left(i+2\right)}l_{i+2}} & -\left(\frac{\beta_{i+1}+k_{2\left(i+1\right)}^{2}}{k_{2\left(i+1\right)}}\right)e^{k_{2\left(i+2\right)}l_{i+2}}\\ E_{i+2}I_{i+2}\left(\beta_{i+2}+k_{1\left(i+2\right)}^{2}\right)e^{k_{1\left(i+2\right)}l_{i+2}} & E_{i+2}I_{i+2}\left(\beta_{i+2}+k_{1\left(i+2\right)}^{2}\right)e^{-k_{1\left(i+2\right)}l_{i+2}} & E_{i+2}I_{i+2}\left(\beta_{i+2}+k_{2\left(i+2\right)}^{2}\right)e^{k_{2\left(i+2\right)}l_{i+2}} & E_{i+2}I_{i+2}\left(\beta_{i+2}+k_{2\left(i+2\right)}^{2}\right)e^{-k_{2\left(i+2\right)}l_{i+2}}\\ -\left(\frac{\kappa_{i+1}G_{i+1}S_{i+1}\beta_{i+1}}{k_{1\left(i+1\right)}}\right)e^{k_{1\left(i+2\right)}l_{i+2}} & \left(\frac{\kappa_{i+1}G_{i+1}S_{i+1}\beta_{i+1}}{k_{1\left(i+1\right)}}\right)e^{-k_{1\left(i+2\right)}l_{i+2}} & -\left(\frac{\kappa_{i+1}G_{i+1}S_{i+1}\beta_{i+1}}{k_{2\left(i+1\right)}}\right)e^{k_{2\left(i+2\right)}l_{i+2}} & \left(\frac{\kappa_{i+1}G_{i+1}S_{i+1}\beta_{i+1}}{k_{2\left(i+1\right)}}\right)e^{-k_{2\left(i+2\right)}l_{i+2}} \end{array}\right] \end{array}$$

By integrating Equations 9b and 12b,

(14) $$\begin{array}{ccc} & & T_{i,i+1\left(i\right)}^{-1}T_{i,i+1\left(i+1\right)}T_{i+1,i+2\left(i+1\right)}^{-1}T_{i+1,i+2\left(i+2\right)}{\left[\begin{array}{c} A\\ B\\ C\\ D \end{array}\right]}_{i+2}\\ & & =B_{i,i+2\left(i\right)}^{-1}e^{-\mathrm{jka}}B_{i,i+2\left(i+2\right)}{\left[\begin{array}{c} A\\ B\\ C\\ D \end{array}\right]}_{i+2} \end{array}$$

For obtaining a non‐trivial solution,

(15) $$\begin{array}{c} \det\;\left|\;T_{i,i+1\left(i\right)}^{-1}T_{i,i+1\left(i+1\right)}T_{i+1,i+2\left(i+1\right)}^{-1}T_{i+1,i+2\left(i+2\right)}-B_{i,i+2\left(i\right)}^{-1}e^{-\mathrm{jka}}B_{i,i+2\left(i+2\right)}\right|=0 \end{array}$$

Consequently, we analytically derived the relationship between *k* and *ω*, i.e., the frequency dispersion relation. Using the dispersion relation, we formulated a band structure to analyze the wave physics of the proposed TREM comprehensively. Moreover, to validate the superiority of the Timoshenko–Ehrenfest beam theory employed in this study, we analyzed the proposed TREM using the traditional exploited Euler–Bernoulli beam theory (Figure S1, Supporting Information) and numerically calculated the results using the finite element method.

## 3 Multifunctional Wave Manipulation

Following the analytically calculated dispersion relation, we illustrate the band structure for the proposed unit cell of the TREM across various values of *h*_m_ along a 1D Bloch wavenumber; *h*_m_ =  2,  5,  8,  11,  and 14 mm (Figure 2b,c). To ensure the propagation of the flexural wave as the primary wave mode, we determined a sufficiently thin plate thickness of *h*  =  2 mm. The lengths of beam sections constituting the unit cell were set as *l*_1_ = *l*_3_  =  2.5 mm and *l*_2_ =  5 mm, whereas the beam widths were designated as *b*_1_ = *b*_3_  =  10 mm and *b*_2_ =  18 mm. The lattice constant, *a*  = *l*_1_  + *l*_2_ +  *l*_3_ =  10 mm was significantly shorter than the wavelength of interest; thus, it satisfied the subwavelength condition. By exploiting the Kirchhoff–Love thin plate theory^[^ ^39^ ^]^ at an operating frequency of 5 kHz, the corresponding wavelength was 62.3 mm (Figure S2, Supporting Information). To facilitate the interconnection of the assembly‐components on the plate, after setting the hole diameter equal to the pillar diameter of the assembly‐components, *d*_p_ =  3 mm, we calculated the dispersion relation while progressively varying *h*_m_ from zero to 14 mm. Consequently, examining the outcomes obtained through the Timoshenko–Ehrenfest beam theory revealed that, with an increasing *h*_m_, the band structure of the flexural wave flattens, implying a decrease in the phase velocity of the elastic wave. This is because of the increased tendency of the unit cells to locally resonate and absorb the propagating wave energy as *h*_m_ increases. We confirmed a remarkable consistency of these results when compared to the numerically calculated results using the finite element method; for *h*_m_ =  11 mm, which is sufficient to cover the full range of the phase‐shift, we observed that the cutoff frequency obtained through the Timoshenko–Ehrenfest beam theory, 5.14 kHz, almost perfectly matched the cutoff frequency obtained through the finite element method, 5.66 kHz. In contrast, the analytically calculated results obtained using the Euler–Bernoulli beam theory showed a significant discrepancy when compared to the previously mentioned results. For *h*_m_ =  11 mm, the cutoff frequency of the flexural wave was completely incorrect at 24.94 kHz. This discrepancy existed because the slenderness ratio of the unit cells comprising the metasurface was close to one, making shear deformation and rotational inertia the dominant factors. However, when the slenderness ratio exceeded 10, the trends in both analytical approaches were aligned (Figure S3, Supporting Information). Consequently, we verified that the results obtained using our proposed Timoshenko–Ehrenfest beam theory‐based analytical model were consistent with the numerically calculated results.

### 3.1 Phase‐Shift

**Figure 3:**
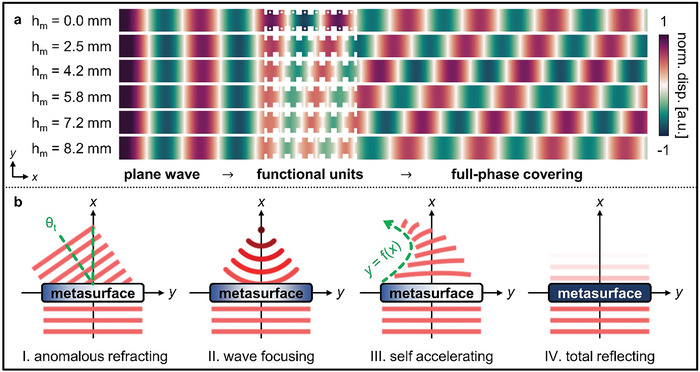
a) Numerically calculated transverse displacement fields in the meta‐slab varying with different values of *h*_m_ at 5 kHz. b) Multifunctional wave manipulating methods based on the generalized Snell's law.

To understand how the results from the previous dispersion relation analysis manifest the wave behavior in the frequency domain, we conducted an analysis of the meta‐slabs through full‐wave harmonic simulations (Figure S4, Supporting Information). We systematically varied *h*_m_ over an ultrabroadband frequency range of 1–20 kHz and examined the transmission of the transmitted waves (Figure 2d). In the passband region of the flexural wave, the wave was transmitted with a high transmission ratio across all the calculated frequency ranges, whereas in the bandgap region, the transmission converged to zero. In this study, meta‐slabs consist of 8‐unit cells. This choice was made because the calculated maximum transmission was found to be the highest, at 81.5%, in an array of 8‐unit cells for varying *h*_m_ (Figure S5, Supporting Information). Moreover, for an identical *h*_m_, once the frequency exceeded the second onset frequency where the bandgap ended, the wave phenomena of the second‐order asymmetric Lamb wave became apparent. However, because this study primarily focused on understanding the behavior of the flexural wave, the first‐order asymmetric Lamb wave and phase‐shifts were calculated for the frequency range below the first bandgap (Figure 2e). This implies that the TREM exhibits an ultra‐broadband frequency range with high‐transmission capabilities, thus, enabling phase‐shift adjustments for reconfigurability and wavefront modulation. To further validate the wave phenomena, we demonstrated the results of varying *h*_m_ at 5 kHz, which resulted in phase‐shifts in the transmitted wave (**Figure** **3**a). According to Fermat's principle, these wavefront modulations enable the multifunctionality of a single substrate. In this study, we validated four distinct unnatural wave phenomena by exploiting the generalized Snell's law: anomalous refraction, focusing, self‐acceleration, and total reflection (Figure 3b).

### 3.2 Anomalous Refraction

**Figure 4:**
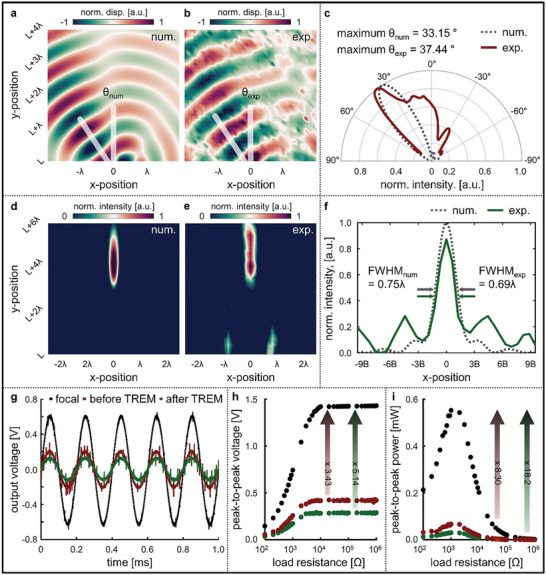
a) Numerically calculated and b) experimentally scanned results of the transverse displacement fields for anomalous wave refraction at 5 kHz, and c) radiation patterns for each result. d) Numerically calculated and e) experimentally scanned results of the transverse intensity fields for a wave focusing at 5 kHz. f) The horizontal intensity passing through the desired focal length of 5*λ*. g) Piezoelectric voltage signal measured from the Timoshenko–Ehrenfest beam‐based reconfigurable elastic metasurface (TREM) configured for a wave focusing at the open‐circuit condition. h) Peak‐to‐peak voltage measured with varying load resistance and i) the corresponding output power.

As the first case of multifunctional wavefront modulation using a TREM, we implemented an anomalous refraction of incident flexural waves. By arranging the meta‐slabs with a specific phase‐shift at regular intervals of 20 mm, we can analytically calculate the angle of refraction based on the generalized Snell's law as follows:

(16) $$\frac{\sin\left(\theta_{t}\right)}{\lambda_{t}}-\frac{\sin\left(\theta_{i}\right)}{\lambda_{i}}=\frac{1}{2\pi }\frac{d\psi \left(y\right)}{\mathrm{dy}}$$

where *λ*_*i*_ and *λ*_*t*_ represent the incident and refraction wavelengths, which are determined by the plate thickness obeying the Kirchhoff–Love plate theory; *θ*_*i*_ and *θ*_*t*_ represent the incident and refraction angles. We can determine *θ*_*t*_ from Equation 16 because we calculated phase‐shift *d* *ψ*(*y*) as a function of *h*_m_ between two adjacent meta‐slabs. Then, we validated anomalous refraction by arranging six meta‐slabs, each contributing a phase‐shift of 2π/6 to cover the full phase‐shift range: *h*_m_ = 0.0, 2.5, 4.2, 5.8, 7.2, and 8.2 mm. According to Equation 16, for a normally incident wave, the theoretical refraction angle for this configuration is $\theta_{t}\left(\frac{2\pi }{6}\right)\;=\;31.28^{\circ }$ at 5 kHz. By analyzing the transverse displacement field obtained through a numerical simulation, we confirmed that a normally incident wave exhibited steering with a maximum refraction angle of 33.15° (**Figure** **4**a,c). Subsequently, we experimentally verified this; for an identical phase distribution, the radiation pattern revealed a maximum refraction angle of 37.44°, which closely aligns with the theoretical and numerical results, demonstrating the anomalous refracting‐wave phenomenon (Figure 4b,c). Moreover, the refraction angle depended on the number of meta‐slabs arranged. Therefore, we compared various numbers of meta‐slabs to observe the change in the refraction angle (Figure S8, Supporting Information). As the number of meta‐slabs increased from four to eight, the refraction angle varied from 51.15° to 22.92°. Furthermore, we conducted numerical simulations for anomalous refraction from 3 to 15 kHz to validate the ability of the proposed TREM to demonstrate wavefront modulation characteristics in the ultrabroadband frequency range. We observed various refraction angles ranging from 42.32° to 17.51° depending on the operating wavelength (Figure S9, Supporting Information).

### 3.3 Wave Focusing

We validated the wave‐focusing ability as a multifunctional TREM. Elastic wave focusing is one of the most important characteristics of wavefront modulation and can be effectively applied in versatile applications, such as energy harvesting and signal sensing. To realize wave‐focusing functionality using the TREM, we reconfigured the TREM with 19 meta‐slabs. The phase‐shifts associated with each meta‐slab are defined according to the generalized Snell's law as follows:

(17) $$\psi \left(y\right)=\frac{2\pi }{\lambda_{t}}\left\{\sqrt{f_{x}^{2}+{\left(y-f_{y}\right)}^{2}}-f_{x}\right\}$$

where the focal position is denoted as (*f_x_*, *f_y_*). Consequently, we calculated the phase distribution that determined the focusing behavior at the desired focal point using Equation 17. As a specific case at 5 kHz, we achieved the phase distribution for the focal position of (5*λ*,  0) =  (0.31,  0) m, from which we obtained the corresponding *h*_m_ = 0.0, 2.1, 3.4, 4.5, 5.4, 6.1, 6.6, 6.8, 7.0, and 7.1 mm with axis symmetry. In this configuration, we calculated the transverse intensity field obtained through a full‐wave harmonic simulation (Figure 4d). We observed that planar incident waves passing through the TREM region exhibited a maximum intensity near the desired focal point of (0.33,  0) m. Moreover, we experimentally validated this phenomenon, and the maximum value of the intensity field was captured at (0.33,  0) m, which closely matched the designed focal point and simulated results (Figure 4e). To quantitatively assess the focusing ability of the TREM, the intensity of the horizontal line was obtained from the numerically and experimentally measured results (Figure 4f). Each result shows the remarkable focusing ability by comparing the full‐width at half maximum (FWHM) values: FWHM from the numerical results was 0.75*λ*, and that from the experimental results was 0.69*λ*, which satisfied the subwavelength focusing ability. The normalized intensity distributions are also demonstrated with the Strehl ratio of 0.87, indicating that the TREM effectively focuses the elastic wave. A slight discrepancy between the two results may have occurred due to factors such as material damping loss and tolerances in the fabricated TREM. However, the numerical results provide sufficient validity to support the experimental results.

The versatility of the TREM enabled its application to various scenarios. As an example of one application, we experimentally verified high‐performance piezoelectric energy harvesting.^[^ ^40^, ^41^ ^]^ In this case, the TREM was used to focus and confine the elastic wave energy at the desired focal point, converting it into enhanced electrical energy from the attached piezoelectric elements. For the piezoelectric energy harvester, we employed piezoelectric elements identical to those used to generate the incident wave. The concentrated strain energy resulted in enhanced electrical energy generation through the piezoelectric effect (Figure S10, Supporting Information). To compare the extracted electrical energy at the focal position, we attached piezoelectric energy harvesters both in front of and behind the TREM and detected the voltage signals at three different locations. Under open‐circuit conditions, the voltage signal exhibited the same waveform as the continuous sinusoidal wave transmitted by the incident wave (Figure 4g). The peak‐to‐peak voltages at the focal position and before and after the TREM were measured at 1.44, 0.42, and 0.28 V, respectively. Because of the decreased transmission while passing through the TREM, the voltage signal detected after the TREM was below that before the TREM. However, the voltage signal detected at the focal position was 3.43 times higher than that before the TREM and an impressive 5.14 times higher than that after the TREM, demonstrating its superiority as a high‐performance piezoelectric energy‐harvesting platform. Furthermore, the load resistance of the electrical circuit was varied to determine the optimal electrical performance (Figure 4h). At ≈5 kΩ, the voltage signal converged to the values measured under open‐circuit conditions. The output power calculated according to Ohm's law was optimal at ≈1 kΩ, and the output power at the focal position was extracted at a significantly improved value, being 8.30 and 18.2 times higher than before and after the TREM, respectively (Figure 4i). Additionally, in real‐world applications, the maintenance of wave focusing ability across a broadband frequency range is also a crucial factor. Therefore, we demonstrated the excellent wave focusing ability by analyzing it across a broadband frequency range with the identical configuration of TREM (Figure S11, Supporting Information). Moreover, for optimal focusing ability, we validated the capability of focusing waves at an identical focal point in the ultrabroadband frequency range by reconfiguring TREM according to the phase‐shift distribution calculated for the target frequency (Figure S12, Supporting Information). In contrast, the reconfigurable ability of the TREM enables flexible modulation of the focal position, making it a more practical platform for piezoelectric energy harvesting. To validate this, we systematically adjusted the focal position vertically (Figure S13, Supporting Information) and horizontally (Figure S14, Supporting Information), thus, confirm the concentration of the intensity at the desired focal position.

### 3.4 Self‐Acceleration

Self‐acceleration, which is a method for guiding transmitted elastic waves along desired trajectories, is an intriguing wave‐control technique. This approach enables elastic wave energy to propagate, thereby avoiding obstacles and complex structures. According to the generalized Snell's law in Equation 16, combining with $\sin\left(\theta_{t}\right)=y^{\prime }\left(x\right)/\sqrt{1+y^{\prime }{\left(x\right)}^{2}}\;\;\;$, the phase‐shift distribution of self‐accelerating trajectory can be expressed as:^[^ ^25^ ^]^

(18) $$\frac{d\psi \left(y\right)}{\mathrm{dy}}=\frac{2\pi }{\lambda_{t}}\frac{y^{\prime }\left(x\right)}{\sqrt{1+y^{\prime }{\left(x\right)}^{2}}}$$

In this study, we applied parabolic and semi‐circular paths to bend the transmitted waves. First, the function of the parabolic trajectory is given by $y\left(x\right)=\sqrt{\alpha x}\;\;\;$, where the constant α determines the degree of wave bending. According to the Equation 18, the phase‐shift distribution is formulated as follows:

(19) $$\psi \;\left(y\right)=\frac{2\pi }{\lambda_{t}}\left[-\frac{\alpha }{4}\ln\left\{y+\sqrt{y^{2}+{\left(\frac{\alpha }{4}\right)}^{2}}\right\}\right]$$

**Figure 5:**
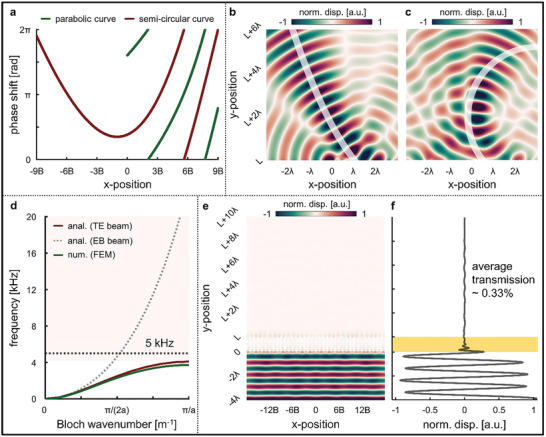
a) Phase‐shift distribution for the self‐acceleration of the transmitted wave. Transverse displacement field depicting the wave phenomena propagating along trajectories defined by b) parabolic and c) semi‐circular functions. d) Band structures calculated for *h*_m_ = 14 mm for each method. The pink‐shaded region represents the bandgap frequency range for flexural wave mode. e) Transverse displacement field demonstrating the total reflecting phenomenon of flexural waves not passing through the Timoshenko–Ehrenfest beam‐based reconfigurable elastic metasurface (TREM) at 5 kHz, and f) the corresponding displacement along the vertical line. The yellow‐shaded region represents the TREM region.

We calculated the phase‐shift distribution for a trajectory with *α*  =  0.20 (**Figure** **5**a) and conducted full‐wave harmonic simulations at 5 kHz for a metasurface composed of 10 meta‐slabs. From the transverse displacement field, we confirmed that the incident planar wave was focused and propagated along the designated bent path (Figure 5b). Furthermore, to validate the control of the bending degree by adjusting the value of α, we varied α from 0.05 to 0.40, guiding the transmitted flexural wave along the desired trajectory (Figure S15, Supporting Information). For the other case, we examined the wave‐bending performance for the semi‐circular function, which is given by $y\left(x\right)=\sqrt{r^{2}-{\left(r-x\right)}^{2}}$, and the wave bending characteristic was determined by radius *r*. According to the Equation 18, the phase‐shift distribution for a semicircular trajectory is determined as follows:

(20) $$\psi \left(y\right)=\frac{2\pi }{\lambda_{t}}\left\{y-2r\tan^{-1}\left(\frac{y}{r}\right)\right\}+C$$

where *C* is an integration constant derived from generalized Snell's law. When a 5 kHz incident planar wave was applied to the TREM composed of 19 meta‐slabs and then reconfigured for the case with *r*  =  0.20, there was the transmitted wave bent along the desired path (Figure 5c). Moreover, we observed that the reconfigured TREMs bent as desired in each case, depending on the variation in *r* from 0.10 to 0.30 (Figure S16, Supporting Information).

### 3.5 Total Reflection

Vibrations stemming from elastic waves are often regarded as factors that must be avoided or mitigated in many cases. As the final aspect of the multifunctionality introduced in our study, we present the concept of total reflection, in which elastic waves cannot propagate through the metasurface region. Based on the previous band analysis of the TREM, we observed a decrease in the cutoff frequency of the flexural waves as *h*_m_ increased. This implies that the assembly‐components with additional masses act as local resonators. To investigate the bandgap behavior, we calculated that the analytical results derived using the Timoshenko–Ehrenfest beam theory for *h*_m_ =  14 mm closely matched the numerically calculated results (Figure 5d). The analytical results indicated a cutoff frequency of 4.09 kHz, whereas the numerical results produced a value of 3.71 kHz. Consequently, the 5 kHz flexural wave used as an example falls within the bandgap region, where flexural waves cannot propagate across the metasurface beyond the cutoff frequency. The slight discrepancy is due to the omission of considerations for longitudinal or torsional wave modes in the analytical beam theory, which are not prominent wave modes in thin plates. However, for an identical *h*_m_, the cutoff frequency obtained through the Euler–Bernoulli beam theory is 22.77 kHz, indicating a significant deviation from the results, rendering it unsuitable as an analytical model. When observing the transverse displacement field in the TREM configured with *h*_m_ =  14 mm, it is evident that no transmitted wave propagated beyond the functional units as the incident planar wave passed through the TREM (Figure 5e). We extracted the transverse displacement for a vertical line ranging from − 4*λ* to *L* + 10*λ* (Figure 5f). Consequently, the average displacement of the transmitted wave was 0.33% that of the incident wave, proving the phenomenon where the majority of the elastic waves fail to propagate the TREM.

## 4 Conclusion

In this study, we investigated a Timoshenko–Ehrenfest beam‐based reconfigurable elastic metasurface (TREM) operating in the ultrabroadband frequency range to achieve multifunctional wave modulation with a high transmission ratio. We theoretically analyzed the dispersion relation governing wave propagation based on the Timoshenko–Ehrenfest beam theory and the transfer matrix method. We validated that our analytical model, which considers shear deformation and rotational inertia, matches well with the numerical and experimental results, compared to the traditional Euler–Bernoulli beam theory. Moreover, we numerically and experimentally verified the multifunctional wave fields within a single substrate by reconfiguring the assembly‐components, including anomalous refraction, focusing, self‐acceleration, and total reflection. First, we verified that the refraction angle of the transmitted wave varies with the number of meta‐slabs and the operating frequency that controls the phase‐shift. Through an experimental visualization of the transmitted wave fields, we confirmed that the designed TREM effectively controlled the wavefront as intended. Second, the TREM was reconfigured to focus on the elastic wave energy at the desired focal point. The experimental results demonstrate the subwavelength focusing ability, confirming the excellence of the TREM. Furthermore, we explored the self‐accelerating wave phenomena propagating along parabolic and semicircular trajectories and investigated the bandgap mechanism leading to the total reflection of the transmitted waves.

In addition, we conducted experiments on piezoelectric energy harvesting, thereby confirming versatile application scenarios. In the TREM configured for wave focusing, we extracted significantly enhanced electrical output power from the piezoelectric element attached to the designated focal position, which was 8.30 and 18.2 times higher than that before and after the TREM, respectively. The various parametric values adopted in this study to design the TREM may be adjusted to match the desired functionality through an appropriate optimization process, or they may be modified to explore richer wave physics, including non‐local effects and topological states. In this process, we believe that the dispersion relation derived from the proposed Timoshenko–Ehrenfest beam theory will provide significant academic guidance and be applied to design various mechanical systems more accurately and effectively. We believe that our analytical modeling of the proposed TREM, based on theoretical beam theory, will provide significant academic guidance not only for metasurfaces but also for designing various mechanical systems. Furthermore, the multifunctional wave manipulating phenomena enabled by our proposed TREM are anticipated to find a wide range of industrial application scenarios in various structures of different scales, such as automobiles, bridges, and ships for versatile functionalities including energy harvesting, structural health monitoring, and Internet of Things.

## 5 Experimental Section

### Numerical Simulations

Throughout this study, all numerical simulations were conducted using the finite element method based on the commercial software COMSOL Multiphysics 6.1, with a physics interface of solid mechanics. The dispersion relations were calculated from the eigenfrequency domain using the Floquet periodic condition, and all full‐wave harmonic simulations were conducted in the frequency domain. To attenuate the reflected wave, all the lateral boundaries were connected to a perfectly matched layer. All components employed in this study were selected from Al‐6061, with mass density, Young's modulus, and Poisson's ratio values of 2700 kg m^−3^, 70 GPa, and 0.33, respectively.

### Fabrication

A 2000 × 1200 × 2 mm^3^ thin aluminum plate, employed as the substrate platform, was fabricated using a laser‐cutting machine to create perforations for the metasurfaces and corresponding holes. The assembly‐components crafted from identical aluminum materials were manufactured using a computer numerical‐control milling machine. All the other materials used in the remaining experiments were purchased commercially.

### Experimental Measurements

To experimentally validate the various wave fields, a continuous sinusoidal incident wave generated by a function generator (33500B, KEYSIGHT) was amplified and refined using a power amplifier (7224, AE TECHRON) to increase the signal‐to‐noise ratio. To generate a plane wave from the 13‐piezoelectric‐transducer array, each transducer with a radius of 20 mm and height of 0.5 mm was aligned with one‐quarter of the wavelength on the substrate plate. Further details and images of the experimental setup (Figure S6, Supporting Information) and planar wave generation (Figure S7, Supporting Information) in free space are provided. To scan the transverse displacement field, a scanning laser Doppler vibrometer (PSV‐400, Polytec) controlled by a data management system (OFV‐5000, Polytec) was used. To examine the piezoelectric energy harvesting performance, the load resistance was adjusted using a resistance substituter (RS‐200, IET Labs), and the measured signal was extracted using an oscilloscope (WaveRunner 610Zi, Teledyne LeCroy).

## Conflict of Interest

The authors declare no conflict of interest.

## Supporting information

Supporting Information

## Data Availability

The data that support the findings of this study are available from the corresponding author upon reasonable request.
