# Supplementary material for: Timoshenko–Ehrenfest Beam‐Based Reconfigurable Elastic Metasurfaces for Multifunctional Wave Manipulation
Source: Adv Sci (Weinh). 2024 Mar 14;11(19):2400090. doi: 10.1002/advs.202400090 (PMC11109653; doi:10.1002/advs.202400090)
Supplement: Supplementary file 1 — Supporting Information [file ADVS-11-2400090-s001.pdf]

ADVANCED SCIENCE
Open Access

Supporting Information

for *Adv. Sci.*, DOI 10.1002/advs.202400090

Timoshenko–Ehrenfest Beam-Based Reconfigurable Elastic Metasurfaces for Multifunctional Wave Manipulation

*Geon Lee, Wonjae Choi, Bonggyu Ji, Miso Kim* and Junsuk Rho**

Supporting Information

**Timoshenko–Ehrenfest Beam-Based Reconfigurable Elastic Metasurfaces for Multifunctional Wave Manipulation**

*Geon Lee, Wonjae Choi, Bonggyu Ji, Miso Kim*, and Junsuk Rho**

G. Lee, Prof. J. Rho
Department of Mechanical Engineering, Pohang University of Science and Technology (POSTECH), Pohang 37673, Republic of Korea
E-mail: jsrho@postech.ac.kr (J. Rho)

Dr. W. Choi, Dr. B. Ji
Intelligent Wave Engineering Team, Korea Research Institute of Standards and Science (KRISS), Daejeon 34113, Republic of Korea

Dr. W. Choi
Department of Precision Measurement, University of Science and Technology (UST), Daejeon 34113, Republic of Korea

Prof. M. Kim
School of Advanced Materials Science and Engineering, Sungkyunkwan University (SKKU), Suwon 16519, Republic of Korea
E-mail: smilekim@skku.edu (M. Kim)

Prof. M. Kim

SKKU Institute of Energy Science and Engineering (SIEST), Sungkyunkwan University (SKKU), Suwon 16519, Republic of Korea

E-mail: smilekim@skku.edu (M. Kim)

Prof. J. Rho

Department of Chemical Engineering, Pohang University of Science and Technology (POSTECH), Pohang 37673, Republic of Korea

E-mail: jsrho@postech.ac.kr (J. Rho)

Prof. J. Rho

Department of Electrical Engineering, Pohang University of Science and Technology (POSTECH), Pohang 37673, Republic of Korea

E-mail: jsrho@postech.ac.kr (J. Rho)

Prof. J. Rho

POSCH-POSTECH-RIST Convergence Research Center for Flat Optics and Metaphotonics, Pohang 37673, Republic of Korea

E-mail: jsrho@postech.ac.kr (J. Rho)

Keywords: elastic metasurface; reconfigurability and multifunctionality; Timoshenko-Ehrenfest beam theory; transfer matrix method; generalized Snell's law

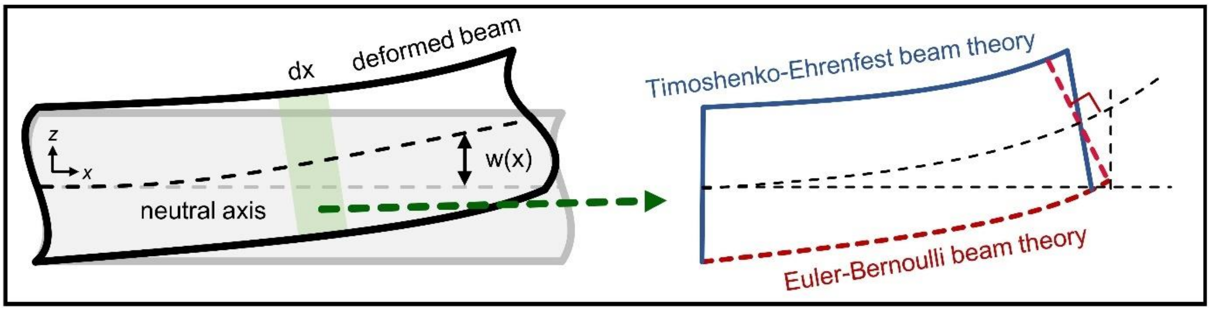

**Figure S1**. Schematic of the comparison between the Timoshenko–Ehrenfest beam theory and the Euler-Bernoulli beam theory.

To demonstrate the superiority of our proposed Timoshenko–Ehrenfest (TE) beam theory-based analytic approach, we compared it with the Euler–Bernoulli (EB) beam theory, which does not consider shear deformation and rotational inertia. For linear elastic, isotropic, and homogeneous EB beams, the equation of motion is derived as follows:

$$E_i I_i \frac{\partial^4 w_i(x,t)}{\partial x^4} + \rho_i S_i \frac{\partial^2 w_i(x,t)}{\partial t^2} = 0. \quad \text{(S1)}$$

Then, we considered the harmonic traveling wave as:

$$w_i(x,t) = W_i e^{j(k_i x - \omega_i t)}. \quad \text{(S2)}$$

By substituting **Equations S1** and **S2**, we obtain the frequency dispersion relation of the EB beam as follows:

$$\omega_i = k_i^2 \sqrt{\frac{E_i I_i}{\rho_i S_i}}. \quad \text{(S3)}$$

To connect each adjacent beam section of the unit cell, we applied the compatibility conditions:

$$W_i(x_i^-) = W_{i+1}(x_i^+), \quad \text{(S4a)}$$
$$W_i'(x_i^-) = W_{i+1}'(x_i^+), \quad \text{(S4b)}$$
$$E_i I_i W_i''(x_i^-) = E_{i+1} I_{i+1} W_{i+1}''(x_i^+), \quad \text{(S4c)}$$
$$E_i I_i W_i'''(x_i^-) = E_{i+1} I_{i+1} W_{i+1}'''(x_i^+), \quad \text{(S4d)}$$

where each equation represents the compatibility conditions for the displacement, slope, bending moment, and shear deformation between the adjacent beam sections. Therefore, we derived a transfer matrix $\boldsymbol{T}_i$ that connects the adjacent $i^{th}$ and $(i+1)^{th}$ beam sections as follows:

$$\boldsymbol{T}_i \cdot \begin{bmatrix} A \\ B \\ C \\ D \end{bmatrix}_i =\cdot \begin{bmatrix} A \\ B \\ C \\ D \end{bmatrix}_{i+1}, \quad \text{(S5)}$$

where

$$\boldsymbol{T}_i = \begin{bmatrix} \left(1+\frac{E_iI_i}{E_{i+1}I_{i+1}}\frac{k_i^2}{k_{i+1}^2}\right)\frac{\cos(k_il_i)}{2} & \left(1+\frac{E_iI_i}{E_{i+1}I_{i+1}}\frac{k_i^2}{k_{i+1}^2}\right)\frac{\sin(k_il_i)}{2} & \left(1-\frac{E_iI_i}{E_{i+1}I_{i+1}}\frac{k_i^2}{k_{i+1}^2}\right)\frac{\cosh(k_il_i)}{2} & \left(1-\frac{E_iI_i}{E_{i+1}I_{i+1}}\frac{k_i^2}{k_{i+1}^2}\right)\frac{\sinh(k_il_i)}{2} \\ \left(-\frac{k_i}{k_{i+1}}-\frac{E_iI_i}{E_{i+1}I_{i+1}}\frac{k_i^3}{k_{i+1}^3}\right)\frac{\sin(k_il_i)}{2} & \left(\frac{k_i}{k_{i+1}}+\frac{E_iI_i}{E_{i+1}I_{i+1}}\frac{k_i^3}{k_{i+1}^3}\right)\frac{\cos(k_il_i)}{2} & \left(\frac{k_i}{k_{i+1}}-\frac{E_iI_i}{E_{i+1}I_{i+1}}\frac{k_i^3}{k_{i+1}^3}\right)\frac{\sinh(k_il_i)}{2} & \left(\frac{k_i}{k_{i+1}}-\frac{E_iI_i}{E_{i+1}I_{i+1}}\frac{k_i^3}{k_{i+1}^3}\right)\frac{\cosh(k_il_i)}{2} \\ \left(1-\frac{E_iI_i}{E_{i+1}I_{i+1}}\frac{k_i^2}{k_{i+1}^2}\right)\frac{\cos(k_il_i)}{2} & \left(1-\frac{E_iI_i}{E_{i+1}I_{i+1}}\frac{k_i^2}{k_{i+1}^2}\right)\frac{\sin(k_il_i)}{2} & \left(1+\frac{E_iI_i}{E_{i+1}I_{i+1}}\frac{k_i^2}{k_{i+1}^2}\right)\frac{\cosh(k_il_i)}{2} & \left(1+\frac{E_iI_i}{E_{i+1}I_{i+1}}\frac{k_i^2}{k_{i+1}^2}\right)\frac{\sinh(k_il_i)}{2} \\ \left(-\frac{k_i}{k_{i+1}}+\frac{E_iI_i}{E_{i+1}I_{i+1}}\frac{k_i^3}{k_{i+1}^3}\right)\frac{\sin(k_il_i)}{2} & \left(\frac{k_i}{k_{i+1}}-\frac{E_iI_i}{E_{i+1}I_{i+1}}\frac{k_i^3}{k_{i+1}^3}\right)\frac{\cos(k_il_i)}{2} & \left(\frac{k_i}{k_{i+1}}+\frac{E_iI_i}{E_{i+1}I_{i+1}}\frac{k_i^3}{k_{i+1}^3}\right)\frac{\sinh(k_il_i)}{2} & \left(\frac{k_i}{k_{i+1}}+\frac{E_iI_i}{E_{i+1}I_{i+1}}\frac{k_i^3}{k_{i+1}^3}\right)\frac{\cosh(k_il_i)}{2} \end{bmatrix}, \tag{S6}$$

Next, to investigate the wave physics of the infinitely arrayed unit cells, we applied the Floquet–Bloch boundary conditions at the ends of the unit cells as follows:

$$W_i(0^+)e^{jka} = W_{i+2}(a^-), \tag{S7a}$$

$$W_i'(0^+)e^{jka} = W_{i+2}'(a^-), \tag{S7b}$$

$$E_iI_iW_i''(0^+)e^{jka} = E_{i+2}I_{i+2}W_{i+2}''(a^-), \tag{S7c}$$

$$E_iI_iW_i'''(0^+)e^{jka} = E_{i+2}I_{i+2}W_{i+2}'''(a^-), \tag{S7d}$$

Therefore, we derived boundary matrix $\boldsymbol{B}$ that connects the adjacent $i^{th}$ and $(i+2)^{th}$ periodic unit beam sections as follows:

$$\boldsymbol{B}_i \cdot \begin{bmatrix} A \\ B \\ C \\ D \end{bmatrix}_{i+2} =\cdot \begin{bmatrix} A \\ B \\ C \\ D \end{bmatrix}_i, \tag{S8}$$

where

$$\boldsymbol{B}_i = \begin{bmatrix} e^{-jka}\left(1+\frac{k_{i+2}^2}{k_i^2}\right)\frac{\cos(k_{i+2}l_{i+2})}{2} & e^{-jka}\left(1+\frac{k_{i+2}^2}{k_i^2}\right)\frac{\sin(k_{i+2}l_{i+2})}{2} & e^{-jka}\left(1-\frac{k_{i+2}^2}{k_i^2}\right)\frac{\cosh(k_{i+2}l_{i+2})}{2} & e^{-jka}\left(1-\frac{k_{i+2}^2}{k_i^2}\right)\frac{\sinh(k_{i+2}l_{i+2})}{2} \\ e^{-jka}\left(-\frac{k_{i+2}}{k_i}-\frac{k_{i+2}^3}{k_i^3}\right)\frac{\sin(k_{i+2}l_{i+2})}{2} & e^{-jka}\left(\frac{k_{i+2}}{k_i}+\frac{k_{i+2}^3}{k_i^3}\right)\frac{\cos(k_{i+2}l_{i+2})}{2} & e^{-jka}\left(\frac{k_{i+2}}{k_i}-\frac{k_{i+2}^3}{k_i^3}\right)\frac{\sinh(k_{i+2}l_{i+2})}{2} & e^{-jka}\left(\frac{k_{i+2}}{k_i}-\frac{k_{i+2}^3}{k_i^3}\right)\frac{\cosh(k_{i+2}l_{i+2})}{2} \\ e^{-jka}\left(1-\frac{k_{i+2}^2}{k_i^2}\right)\frac{\cos(k_{i+2}l_{i+2})}{2} & e^{-jka}\left(1-\frac{k_{i+2}^2}{k_i^2}\right)\frac{\sin(k_{i+2}l_{i+2})}{2} & e^{-jka}\left(1+\frac{k_{i+2}^2}{k_i^2}\right)\frac{\cosh(k_{i+2}l_{i+2})}{2} & e^{-jka}\left(1+\frac{k_{i+2}^2}{k_i^2}\right)\frac{\sinh(k_{i+2}l_{i+2})}{2} \\ e^{-jka}\left(-\frac{k_{i+2}}{k_i}+\frac{k_{i+2}^3}{k_i^3}\right)\frac{\sin(k_{i+2}l_{i+2})}{2} & e^{-jka}\left(\frac{k_{i+2}}{k_i}-\frac{k_{i+2}^3}{k_i^3}\right)\frac{\cos(k_{i+2}l_{i+2})}{2} & e^{-jka}\left(\frac{k_{i+2}}{k_i}+\frac{k_{i+2}^3}{k_i^3}\right)\frac{\sinh(k_{i+2}l_{i+2})}{2} & e^{-jka}\left(\frac{k_{i+2}}{k_i}+\frac{k_{i+2}^3}{k_i^3}\right)\frac{\cosh(k_{i+2}l_{i+2})}{2} \end{bmatrix}, \tag{S9}$$

By integrating Equation **S5** and **S9**,

$$\begin{bmatrix} A \\ B \\ C \\ D \end{bmatrix}_{i+2} = \boldsymbol{B}_i^{-1} \cdot \begin{bmatrix} A \\ B \\ C \\ D \end{bmatrix}_i = \boldsymbol{T}_{i+1} \cdot \boldsymbol{T}_i \cdot \begin{bmatrix} A \\ B \\ C \\ D \end{bmatrix}_i. \tag{S10}$$

For obtaining a non-trivial solution,

$$\det\left|\boldsymbol{B}_i^{-1} - \boldsymbol{T}_{i+1} \cdot \boldsymbol{T}_i\right| = 0. \tag{S11}$$

Consequently, we analytically derived the relationship between $k$ and $\omega$, i.e., the frequency dispersion relation based on the EB beam theory. The TE beam theory is considered equivalent to the EB beam theory, which is a valid approximation when the condition $(3EI)/(\kappa L^2 SG) \ll 1$, and the EB beam theory is recovered in the limit as $\kappa GS \rightarrow \infty$ and $\rho I \rightarrow 0$.

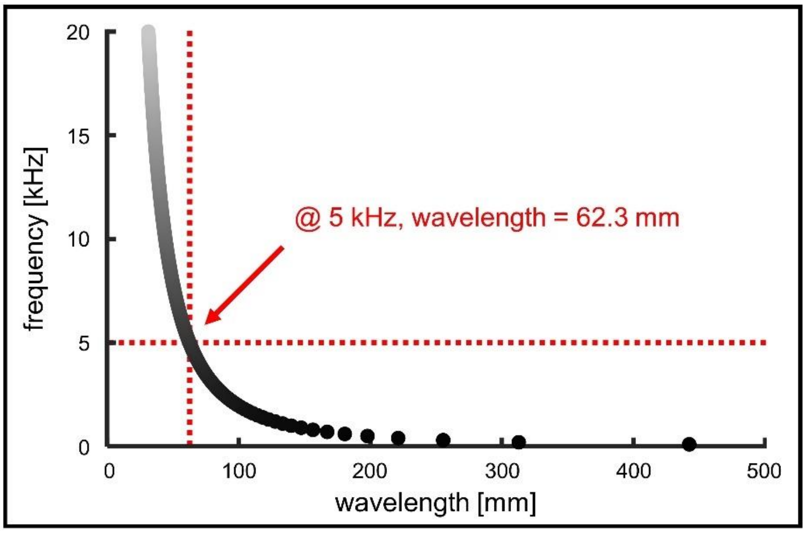


**Figure S2**. Frequency-dependent wavelength variations of flexural waves based on the Kirchhoff–Love plate theory.

To calculate the wavelength of the propagating waves on the thin plate, we employed the Kirchhoff–Love plate theory, which describes the transverse displacement behavior, whose governing equation is defined as follows:

$$\frac{h^3 E}{12(1-\nu^2)} \nabla^4 \xi + \rho h \frac{\partial^2 \xi}{\partial t^2} = 0. \tag{S12}$$

By assuming a monochromatic time dependence of the angular frequency $\omega$, we derived a quadratic dispersion relation for the thin plate for planar flexural wave propagation with $k$ for various thicknesses of the plate:

$$k = \left\{\frac{12\rho(1-\nu^2)}{E}\right\}^{\frac{1}{4}} \left(\frac{\omega}{h}\right)^{\frac{1}{2}}. \tag{S13}$$

By expressing the phase velocity of the propagating flexural wave under the linear relation:

$$v_p = \frac{\omega}{k} = \left\{\frac{E}{12\rho(1-\nu^2)}\right\}^{\frac{1}{4}} h^{\frac{1}{2}} \omega^{\frac{1}{2}}, \quad \text{(S14)}$$

we can obtain the wavelength $\lambda$ under the relation as:

$$\lambda = \frac{v_p}{2\pi\omega}. \quad \text{(S15)}$$

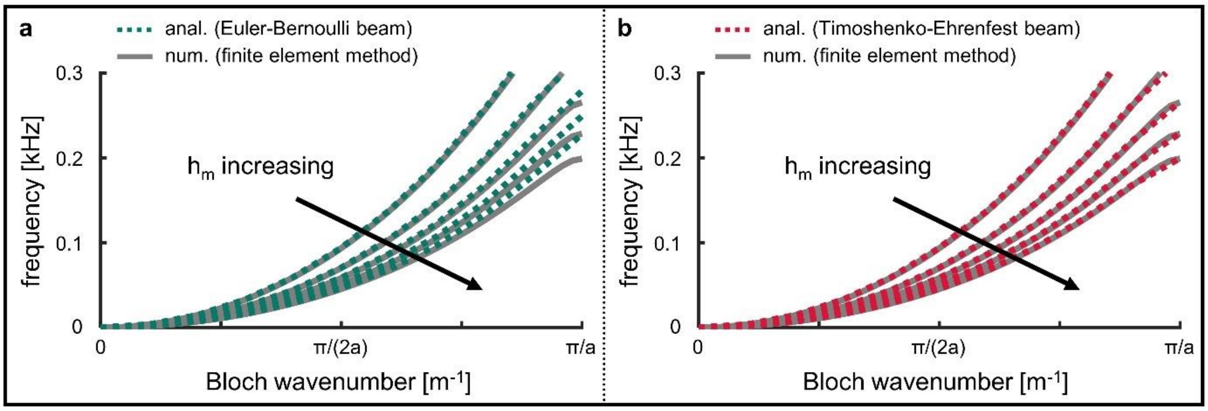


**Figure S3.** Dispersion curves for the high slenderness ratio beam based on the (a) Euler–Bernoulli (EB) beam theory and (c) Timoshenko–Ehrenfest beam theory compared with the numerically calculated results using the finite element method by varying $h_{\mathrm{m}}$.

We calculated the dispersion curves for a high slenderness ratio, where the length/width is ten. The unit cell described in the main manuscript for designing the Timoshenko–Ehrenfest beam-based reconfigurable elastic metasurface (TREM) has a low slenderness ratio. In this case, the results based on the EB beam theory tended to deviate from the trends observed in the TE beam theory and FEM results. However, for a high slenderness ratio, it can be assumed that the influence of rotational inertia and shear deformation is negligible, generally aligning well with the trends. Nevertheless, even in this scenario, as the wavenumber increases, the cutoff frequency introduces a larger error compared to the TE beam theory. Consequently, for both low and high slenderness ratio, we have demonstrated that our proposed analytic model based on the TE beam theory accurately represents the dispersion relation.

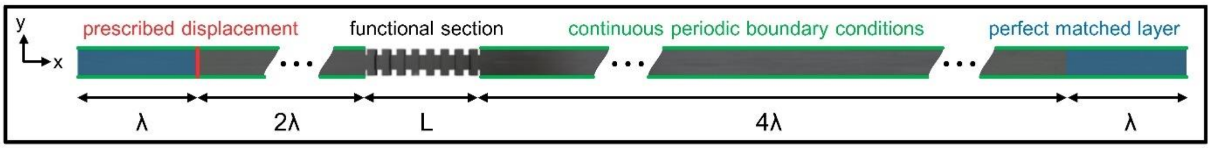


**Figure S4.** Boundary conditions for numerically analyzing the meta-slab.

To investigate the transmitted wave along the TREM, we conducted numerical simulations applying the following boundary conditions to the meta-slab. First, perfect matched layers were set at both ends to eliminate the influence of reflective waves. Then, prescribed displacement induces transverse displacement that propagates as a flexural wave. Continuous periodic boundary conditions were applied to the lateral boundaries of the meta-slab to impart infinite periodicity. Consequently, we systematically reconstructed the TREM to calculate transmission and phase-shift.

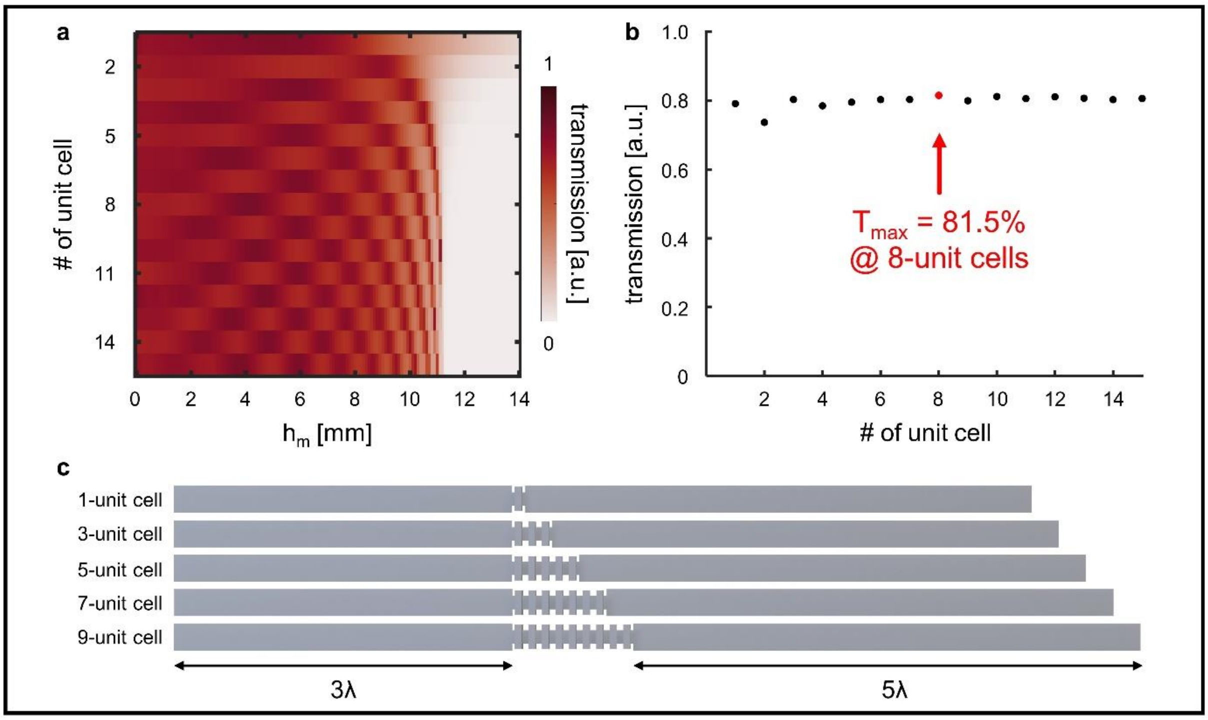


**Figure S5.** (a) Transmission spectrum based on the variation of $h_m$ per number of unit cells, and (b) maximum transmission based on the number of unit cells. (c) Schematic of a meta-slab composed by varying the number of unit cells.

To determine the number of unit cells composing the single meta-slab, we numerically calculated the transmission by varying the $h_{\mathrm{m}}$ with respect to the number of unit cells. In general, regardless of the number of unit cells, the maximum transmission exceeded 80%, but when there were 8-unit cells, the maximum transmission reached its highest value at 81.5%. Consequently, we adopted a meta-slab composed of 8-unit cells. However, even with a reduced number of unit cells to compose the TREM, the proposed TREM can achieve a high transmission ratio. It is also possible to design the total length of functional units to be smaller than the working wavelength. For example, considering a working wavelength of 62.3 mm at 5 kHz, a TREM composed of fewer than 6-unit cells has a total length smaller than the working wavelength with high transmission ratio.

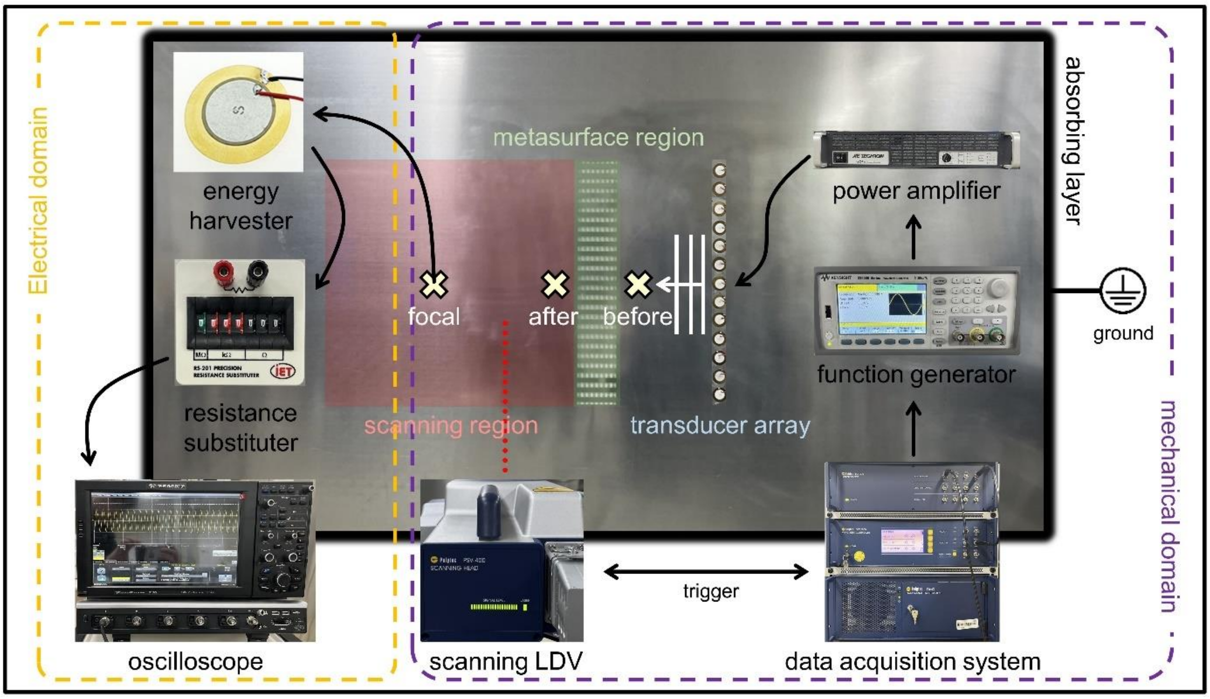


**Figure S6.** Picture of the experimental setup used for verifying the multifunctional wave phenomena of the Timoshenko–Ehrenfest beam-based reconfigurable elastic metasurface and the performance of piezoelectric energy harvesting.

To validate the reconfigurability of TREM and explore multifunctional wave phenomena, experiments were conducted using the following setup. Initially, it is divided into a mechanical domain for generating and measuring elastic waves and an electrical domain for measuring piezoelectric energy harvesting performance. Firstly, to generate an incident wave, a continuous sinusoidal wave signal is generated from a function generator. Subsequently, to enhance the signal-to-noise ratio, the wave signal is refined through a power amplifier, and this electrical signal is then transmitted to a transducer array consisting of 13 piezoelectric disc. To generate a plane wave from the 13-piezoelectric-transducer array, each transducer with a radius of 20 mm and height of 0.5 mm was aligned with one-quarter of the wavelength on the substrate plate. The transducer array generates planar waves through the piezoelectric effect and propagates them through the TREM's domain based on the desired functionality. Consequently, to visualize the behavior of the transmitted wave beyond the TREM domain, a scanning laser Doppler vibrometer was employed. Secondly, the electrical domain for measuring piezoelectric energy harvesting performance, conducted to validate the practicality of the TREM, is built upon the mechanical domain described earlier. The harvesting performance was measured at three positions: before the TREM to assess performance in the incident wave, after the TREM to evaluate performance in the transmitted wave, and at the focal to demonstrate how the confined elastic wave energy enhances electrical power in the configuration designed for wave focusing. Consequently, to determine the maximum output power and corresponding optimal load resistance, the load resistance was varied using a resistance substituter, and the electrical voltage signal extracted through the reverse piezoelectric effect was measured by an oscilloscope.

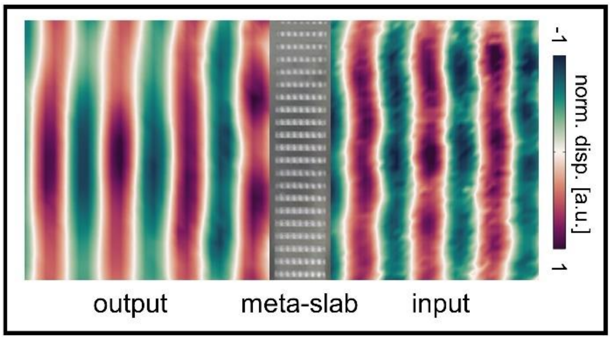


**Figure S7.** Experimentally scanned transverse displacement field of the plane wave generation from the piezoelectric transducer array and the planar transmitted wave without the assembly-components.

To verify the successful formation of a planar wave from the transducer array, we examined the transverse displacement field from the transducer array to the TREM. Additionally, we confirmed the maintained planar wave through the TREM without configuring the assembly-components. Consequently, we confirmed that the transmission of the transmitted wave beyond the TREM is 87.7%, validating the high transmission ratio even as it propagates through the TREM.

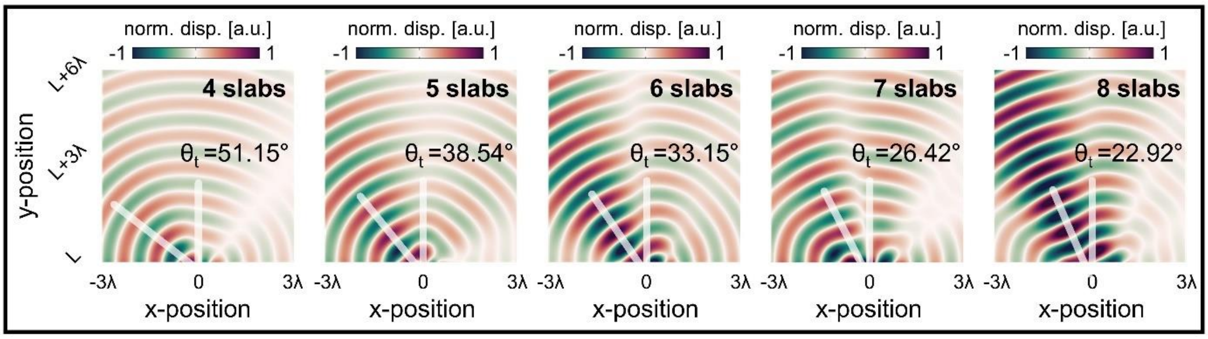


**Figure S8.** Transverse displacement fields demonstrating the variation in the anomalous refraction angle of the transmitted wave with respect to the number of meta-slabs.

To examine the variation in refractive angle with the number of meta-slabs, we numerically calculated the transmitted wave field by arranging meta-slabs from 4 to 8 at 5 kHz. The

refractive angle, calculated by the generalized Snell's law, decreased as the number of meta-slabs increased, and the transverse displacement field exhibited corresponding results. Consequently, we verified the ability to freely manipulate the refractive angle by varying the number of meta-slabs.

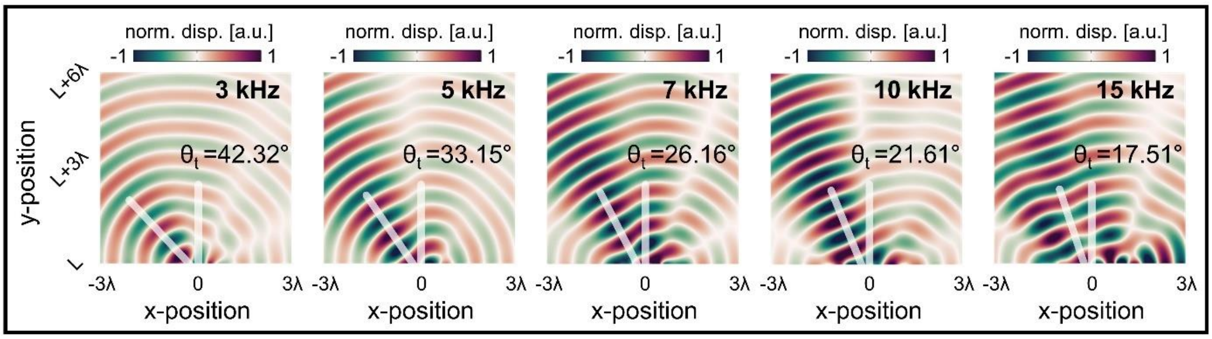


**Figure S9.** Transverse displacement fields indicating the variation in the anomalous refraction angle of the transmitted wave with respect to the ultrabroadband frequency range.

To validate that our designed TREM operates in the ultrabroadband range, we verified the wave refracting ability of the TREM, consisting of 6 meta-slabs, by varying the incident planar wave from 3 to 15 kHz. The wavelength values applied in the generalized Snell's law were calculated based on Kirchhoff–Love plate theory, and the calculated refractive angle aligned with the transmitted wave field. Consequently, we confirmed the potential usage of our TREM for wavefront modulation in the ultrabroad frequency range.

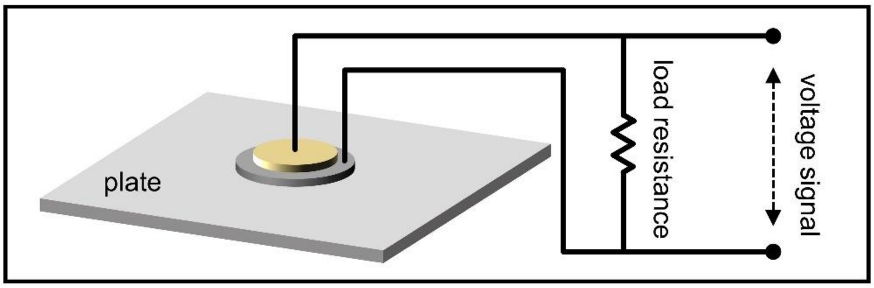


**Figure S10.** Electrical circuit of the piezoelectric energy harvesting system.

For piezoelectric energy harvesting, we described the piezoelectric element and its corresponding electrical circuit. To attach the piezoelectric disc with electrodes on the upper and lower surfaces to a flat aluminum thin plate, the electrode on the lower surface was extended upward. Subsequently, the two electrodes were connected by a resistance substituter, and the electrical voltage signal passing through the load resistance was measured by an oscilloscope.

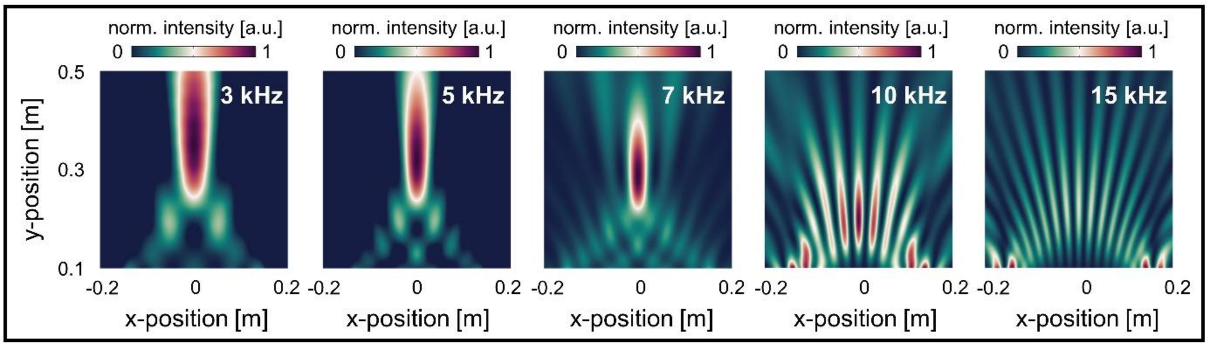


**Figure S11.** Intensity fields in the ultrabroadband frequency range for the elastic metasurfaces configured for wave focusing at 5 kHz.

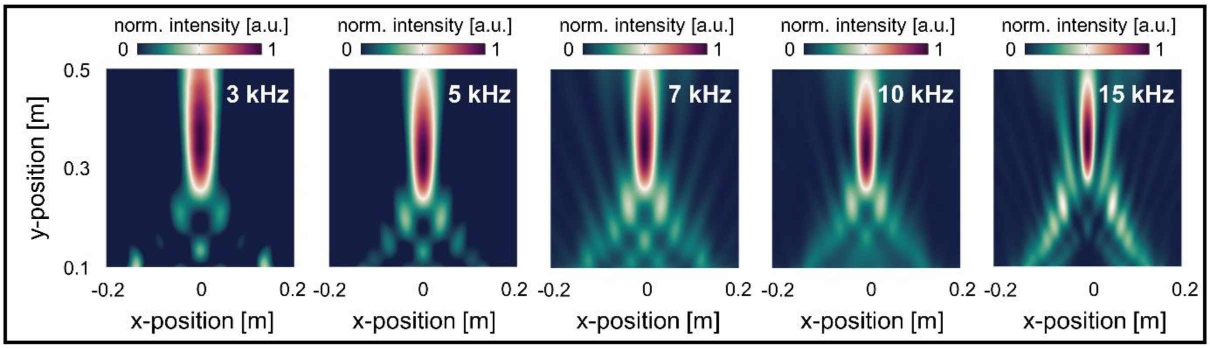


**Figure S12.** Intensity fields in the reconfigurable elastic metasurface at ultrabroadband frequency range at the identical focal position.

The operation of a TREM configured for wave focusing in a broadband frequency range is a crucial factor to consider for various applications. We analyzed whether TREM configured according to the phase-shift distribution calculated for the focal point at (0.3 m, 0 m) at 5 kHz maintains wave focusing ability at different frequencies. As a result, we confirmed the

maintenance of wave focusing ability in the broadband frequency range from 3 kHz to 7 kHz, with slight variations in focal position. However, with the increase in the target frequency, we observed an acceleration in wave scattering. This phenomenon is attributed to the fact that, in the relatively low-frequency range, the dispersion relation tends to be linear. On the other hand, as the frequency increases, the influence of shear deformation and rotational inertia intensifies, leading to a more pronounced dispersive behavior in propagating waves. Nevertheless, these results substantiate that a TREM configured once in the broadband frequency range effectively maintains its capability.

Moreover, in practice, maintaining the location of the focal point at various frequencies is crucial. Therefore, we investigated the confocal wave focusing ability using the reconfigurable capability of TREM across an ultrabroadband frequency range. Performing harmonic simulations for the broadband frequency range, we applied assembly-components corresponding to the phase-shift distribution calculated according to the generalized Snell's law at various frequencies. With the focal point set to (0.3 m, 0 m), we demonstrated the confinement of elastic wave energy at the identical focal position across the ultrabroadband frequency range by visualizing the intensity field.

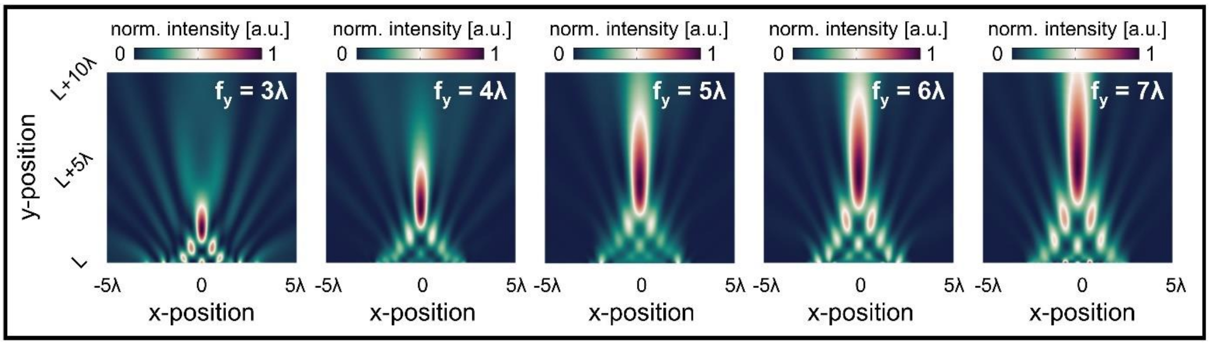


**Figure S13.** Intensity fields in the reconfigurable elastic metasurface with varying focal positions along the vertical axis.

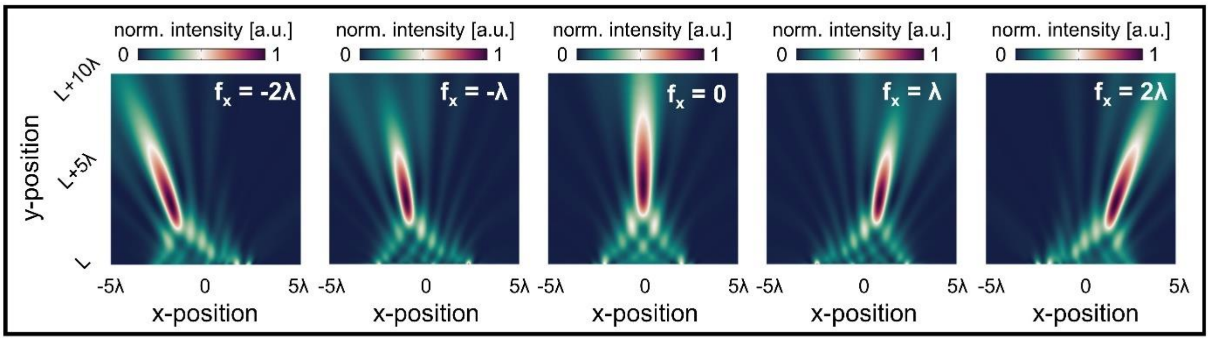


**Figure S14.** Intensity fields in the reconfigurable elastic metasurface with varying focal positions along the horizontal axis.

To verify the controllable manipulation of the focal point through the reconfiguration of the TREM, we obtained intensity fields through numerical simulations by varying the focal position vertically and horizontally. To change the focal position, we calculated the phase-shift distribution based on the generalized Snell's law, confirming the ease with which the focal position can be changed through the reconfigurability of the TREM. Consequently, this ability to vary the focal point suggests the potential to extract high-performance electrical energy tailored to the desired focal position from the perspective of piezoelectric energy harvesting.

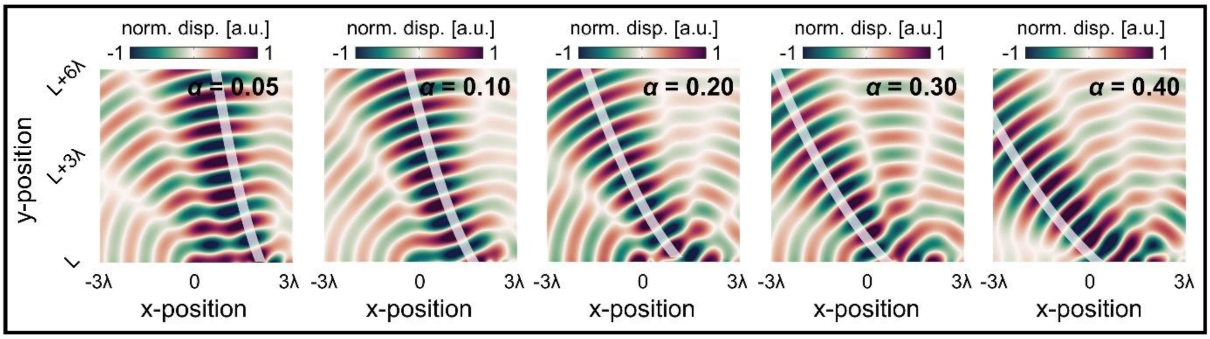


**Figure S15.** Transverse displacement fields for the self-accelerating phenomena propagating along the desired trajectory according to the constant $\alpha$ of the parabolic function.

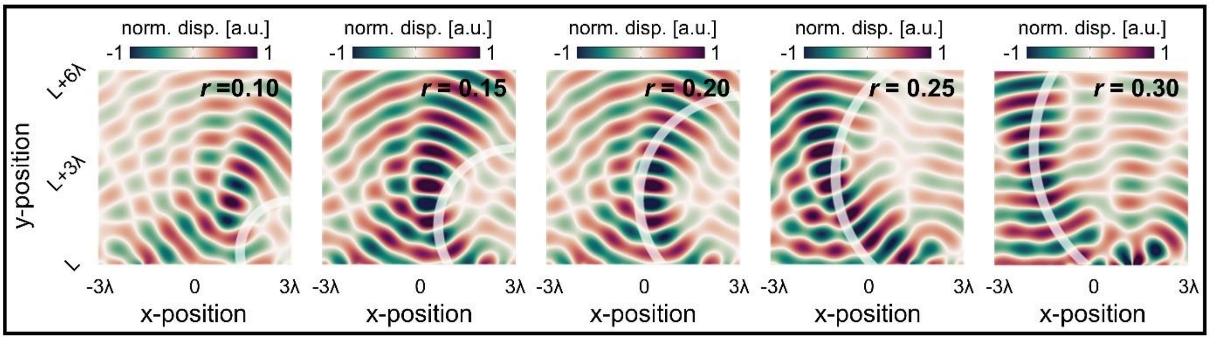


**Figure S16.** Transverse displacement fields for the self-accelerating phenomena propagating along the desired trajectory according to the constant $r$ of the semi-circular function.

To investigate the self-acceleration phenomenon in which waves propagate along the trajectory we defined, we calculated the phase-shift distribution based on the generalized Snell's law according to the desired trajectory function. Subsequently, we reconfigured the TREM based on this information and conducted numerical simulations of the wave field. Consequently, we observed that the degree of wave bending varied depending on the values of the constants $\alpha$ and $r$ for each function, representing the self-acceleration level. We confirmed the propagation of waves along the trajectories of each function, depicted in white-line.
